# Comparative Efficacy and Safety of Glucagon Receptor Agonists on Metabolic Outcomes: A Network Meta‐Analysis of Randomised Controlled Trials

**DOI:** 10.1002/edm2.70187

**Published:** 2026-03-05

**Authors:** Ayah Abulehia, Hazem Ayesh, Omar Ayesh, Doha Jaber, Thekrayat Asad, Adham Itbaisha, Hamzeh Abugharbieh, Raged Gharbia, Lila H. Abu‐Hilal, Barbara Gisella Carranza Leon

**Affiliations:** ^1^ Faculty of Medicine Al‐Quds University Jerusalem Palestine; ^2^ Deaconess Health System Evansville Indiana USA; ^3^ Indiana University School of Medicine Indianapolis Indiana USA; ^4^ Department of Medical Laboratory Sciences Al‐Aqsa University Gaza Palestine; ^5^ Department of Internal Medicine, Unity Hospital, Rochester Regional Health Rochester New York USA; ^6^ Department of Internal Medicine University of Toledo Toledo Ohio USA; ^7^ Department of Internal Medicine, University Hospitals Geauge Medical Center Chardon Ohio USA; ^8^ Division of Diabetes, Endocrinology and Metabolism, Department of Internal Medicine Vanderbilt University Medical Center Nashville Tennessee United States

**Keywords:** body weight, diabetes, glucagon receptor agonists, HbA1c, metabolism, obesity

## Abstract

**Introduction:**

Glucagon receptor agonists (GRAs) are an emerging class of therapies for obesity and type 2 diabetes, demonstrating encouraging metabolic and weight‐reducing effects. Several investigational GRA‐based agents, including retatrutide, cotadutide, mazdutide, and survodutide, have reported promising results across early and mid‐phase clinical trials. This comprehensive meta‐analysis evaluates the efficacy and safety of these agents in individuals with type 2 diabetes, overweight, or obesity.

**Methods:**

PubMed, Cochrane, Embase, and Scopus databases were systematically searched. Fourteen randomised controlled trials meeting the inclusion criteria were analysed using frequentist network meta‐analysis. Random‐effects models were applied to assess mean differences (MD) in weight change, both absolute and percent changes, HbA1c, adverse events, and discontinuation due to adverse events. Heterogeneity was quantified using the *I* ^2^ statistic.

**Results:**

Retatrutide demonstrated the greatest weight reduction versus placebo (MD −13.44 kg; 95% CI [−18.38, −8.51]), followed by survodutide (MD −10.74 kg; 95% CI [−15.68, −5.80]) and mazdutide (MD −6.47 kg; 95% CI [−10.71, −2.24]). Cotadutide showed the smallest and nonsignificant effect (MD −3.41 kg; 95% CI [−11.63, 4.81]). Regarding HbA1c reduction, retatrutide showed the largest effect, followed by survodutide, mazdutide, and cotadutide; however, only the effect of retatrutide reached statistical significance. In terms of safety, mazdutide demonstrated the most favourable tolerability profile, whereas retatrutide and cotadutide were associated with comparatively lower tolerability.

**Conclusions:**

Retatrutide and survodutide exhibit the most favourable efficacy profiles for obesity and T2DM, with acceptable safety. These findings support their potential clinical use and highlight the need for future head‐to‐head trials.

## Graphical abstract

This network meta‐analysis compares emerging glucagon receptor agonists for obesity and type 2 diabetes. Retatrutide and survodutide demonstrated the most favourable efficacy profiles for weight reduction with acceptable safety, supporting their potential as promising therapeutic options and highlighting the need for future head‐to‐head clinical trials.

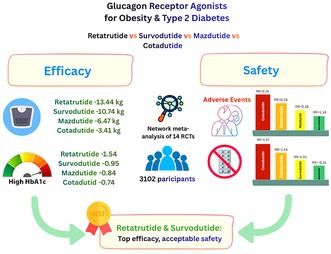

## 1 Introduction

Obesity and type 2 diabetes mellitus (T2DM) are closely interlinked chronic conditions and constitute significant global public health challenges. The World Obesity Atlas 2024 forecasts that by 2035, an estimated 1.53 billion adults will be living with obesity, up from 0.81 [1]. These conditions fundamentally increase the risk of hypertension, dyslipidaemia, cardiovascular disease, and death. Evidence suggests that weight loss of 5%–15% of baseline body weight can significantly reduce these risks and improve metabolic health, making weight reduction a critical therapeutic goal [2].

In recent years, glucagon‐like peptide‐1 receptor agonists (GLP‐1 RAs) have become a cornerstone in the treatment of T2DM and obesity, with agents like semaglutide and liraglutide demonstrating notable efficacy in both weight loss and glycaemic control [3, 4]. In addition to their metabolic benefits, GLP‐1 receptor agonists have clear cardiovascular protective effects. This is shown in major cardiovascular outcome trials, which reported significant reductions in major adverse cardiovascular events [5, 6]. Despite their promising efficacy, dual and triple incretin receptor agonists have been developed to target additional pathways for enhancing metabolic pathways for weight loss and glycaemic control, namely, the glucagon and gastric inhibitory polypeptide (GIP) receptors. ^3,4^ Glucagon receptor agonists (GRAs) work by increasing gluconeogenesis, glycogenolysis, and energy expenditure, promoting lipid oxidation, and improving hepatic fat metabolism, in contrast to the GLP‐1 RAs, which function as appetite‐suppressing and insulinotropic agents [7, 8]. The various mechanisms of action of GRAs add further metabolic advantages beyond those achieved with GLP‐1 RAs alone [8]. Among the recent dual and triple agonists are retatrutide, survodutide, cotadutide, and mazdutide, which have shown significant effects in managing obesity and T2DM.

Multiple randomised controlled trials (RCTs) have investigated the efficacy and safety of retatrutide, survodutide, cotadutide, and mazdutide as treatment options for T2DM, overweight, and obesity. Each study has demonstrated a promising effect of these agents in both diabetes and obesity [4, 9]. Despite these encouraging findings, the evidence base remains fragmented as most studies evaluate each agent independently or compare them only with placebo or established GLP‐1 RAs.

Direct comparisons among these new agents are still limited. Thus, a network meta‐analysis provides a useful way to compare their efficacy and safety indirectly and can guide future clinical decisions [9, 10, 11, 12, 13, 14, 15, 16, 17, 18, 19, 20, 21, 22].

This network meta‐analysis aims to evaluate and compare the efficacy and safety of four emerging dual and triple GRAs: survodutide, cotadutide, retatrutide, and mazdutide, in adults with overweight, obesity, and/or T2DM. The analysis includes RCTs and focuses on key efficacy outcomes such as changes in HbA1c, body weight reduction, and safety outcomes such as the incidence of adverse events and treatment discontinuation rates due to adverse events. By integrating both direct and indirect evidence through a frequentist network meta‐analysis framework, this study seeks to provide a comprehensive comparative assessment of these agents to guide the clinician in decision‐making in managing patients with diabetes and obesity.

## 2 Materials and Methods

### 2.1 Study Design and Registration

This network meta‐analysis was conducted in accordance with the Preferred Reporting Items for Systematic Reviews and Meta‐Analyses (PRISMA) and PRISMA for Network Meta‐Analyses (PRISMA‐NMA) guidelines, see Supplement 10. The study protocol was prospectively registered on the Open Science Framework (OSF) registries (https://osf.io/ek5nu) [23].

### 2.2 Eligibility Criteria

We included RCTs evaluating the efficacy and safety of glucagon receptor agonists, specifically survodutide, cotadutide, retatrutide, and mazdutide, in adult participants (≥ 18 years) diagnosed with obesity (Body Mass Index (BMI) ≥ 30 kg/m [2]), overweight (BMI ≥ 24 kg/m [2]), and/or T2DM. T2DM was defined according to country‐specific or international diagnostic guidelines, such as those of the American Diabetes Association (ADA) or the World Health Organization (WHO), including an HbA1c level ≥ 6.5% [24]. Overweight and obesity were also defined according to the criteria applied in the original trials. Because several included studies used Asian‐specific definitions (overweight: BMI ≥ 24 kg/m [2]), we adopted BMI ≥ 24 kg/m [2] as the lower inclusion threshold to ensure consistency across all populations. Studies were required to report at least one of the following outcomes: primary outcome: mean change in body weight from baseline or versus placebo; secondary outcomes: mean change in HbA1c levels from baseline or versus placebo, and adverse event profiles. Only trials with a minimum follow‐up duration of 12 weeks were included. Studies were excluded if they were non‐randomised or observational, included participants without a formal diagnosis of T2DM, overweight or obesity, investigated interventions other than the specified dual agonists, or lacked complete or accessible outcome data.

### 2.3 Search Strategy

We systematically searched PubMed, Cochrane, Embase, and Scopus to identify relevant RCTs evaluating survodutide, cotadutide, retatrutide, or mazdutide in adults with overweight, obesity, and/or T2DM. The search included combinations of terms related to ‘glucagon receptor agonists’, ‘Survodutide’, ‘Cotadutide’, ‘Retatrutide’, ‘Mazdutide’, ‘obesity’, and ‘type 2 diabetes’. A detailed database‐specific search strategy with full search strings and MeSH terms is provided in Supplement 1. Two reviewers independently screened titles and abstracts, followed by full‐text reviews to identify eligible studies. A third reviewer resolved discrepancies.

### 2.4 Study Selection

**FIGURE 1:**
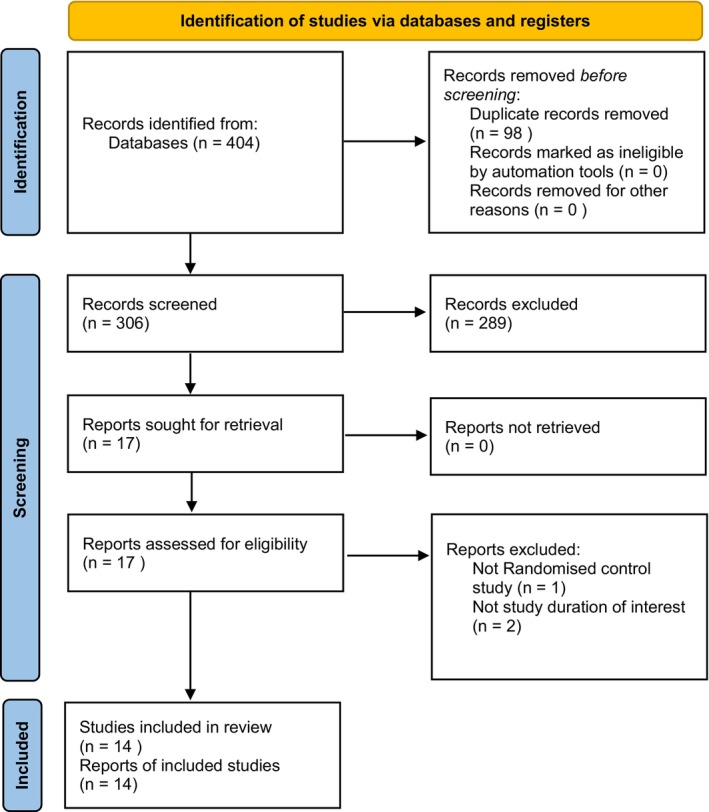
PRISMA flowchart for study selection.

A total of 404 records were identified through database searching (115 Scopus, 25 Embase, 241 Cochrane, and 23 PubMed). A total of 306 studies were included after removing duplicates, 289 were excluded as irrelevant, and the remaining 17 full‐text articles were assessed, with 3 excluded (2 had a short follow‐up period and 1 post hoc study). The remaining 14 papers were considered for final inclusion [9, 10, 11, 12, 13, 14, 15, 16, 17, 18, 19, 20, 21, 22]. The PRISMA flow diagram is displayed in Figure 1.

### 2.5 Data Extraction

Two independent reviewers extracted data using a standardised form. Extracted variables included study characteristics (e.g., sample size, follow‐up duration, and region), patient demographics, and inclusion criteria focusing on patients diagnosed with overweight, obesity, and/or T2DM with at least 12 weeks of follow‐up. Intervention arms included survodutide, cotadutide, retatrutide, or mazdutide compared to placebo. Outcome data included changes in body weight (kg), percent body weight changes, haemoglobin A1c (HbA1c), treatment discontinuation due to adverse events, and rate of adverse events. The risk of bias for each included study was assessed using the Cochrane Risk of Bias (ROB2) tool, rating each domain as low [1], moderate [2], or high [3] risk, see Supplement 9. Additionally, the Confidence in Network Meta‐Analysis (CINeMA) framework was used to evaluate the confidence in this network [25, 26].

### 2.6 Data Synthesis and Statistical Analysis

We conducted a frequentist random‐effects network meta‐analysis (NMA). Mean differences (MDs) with 95% confidence intervals were used for continuous outcomes, while risk ratios (RRs) were used for dichotomous outcomes. Placebo served as the reference comparator in all analyses. For interventions with multiple dosing arms, the highest reported dose for each drug was used in the primary analysis as this approach exhibits real‐world clinical practice where therapies are typically titrated to their maximally tolerated dose. Treatment rankings were estimated using P‐scores (ranging from 0 to 1). The higher P‐score value, which is closer to 1, indicates the most effective treatment among all other remedies.

Heterogeneity across studies was assessed using the I [2] statistic, Tau [2], and Cochran's Q test [27]. Global inconsistency was examined using design‐by‐treatment interaction models. To ensure the validity of the NMA, we assessed the transitivity assumption, which requires that studies are sufficiently comparable in key clinical characteristics to allow valid indirect comparisons through a common comparator. Accordingly, we compared key baseline covariates including age, BMI, and HbA1c levels across treatment comparisons using Analysis of Variance (ANOVA) tests and boxplot visualisations. Meta‐regression analyses were additionally performed to see how differences in baseline characteristics may affect treatment outcomes. However, despite these assessments, the transitivity assumption cannot be fully verified, and some degree of residual clinical heterogeneity may persist. Planned sensitivity analyses included the exclusion of studies at high risk of bias and leave‐one‐out analyses to evaluate the impact of individual studies on the overall network estimates.

A frequentist framework was employed using the netmeta 3.2, R 4.5 package to synthesise both direct and indirect evidence from RCTs evaluating the efficacy, safety, and treatment discontinuation of dual and triple GLP‐1 receptor agonists in adults with obesity and/or T2DM [28].

## 3 Results

### 3.1 Study Selection and Characteristics

**FIGURE 2:**
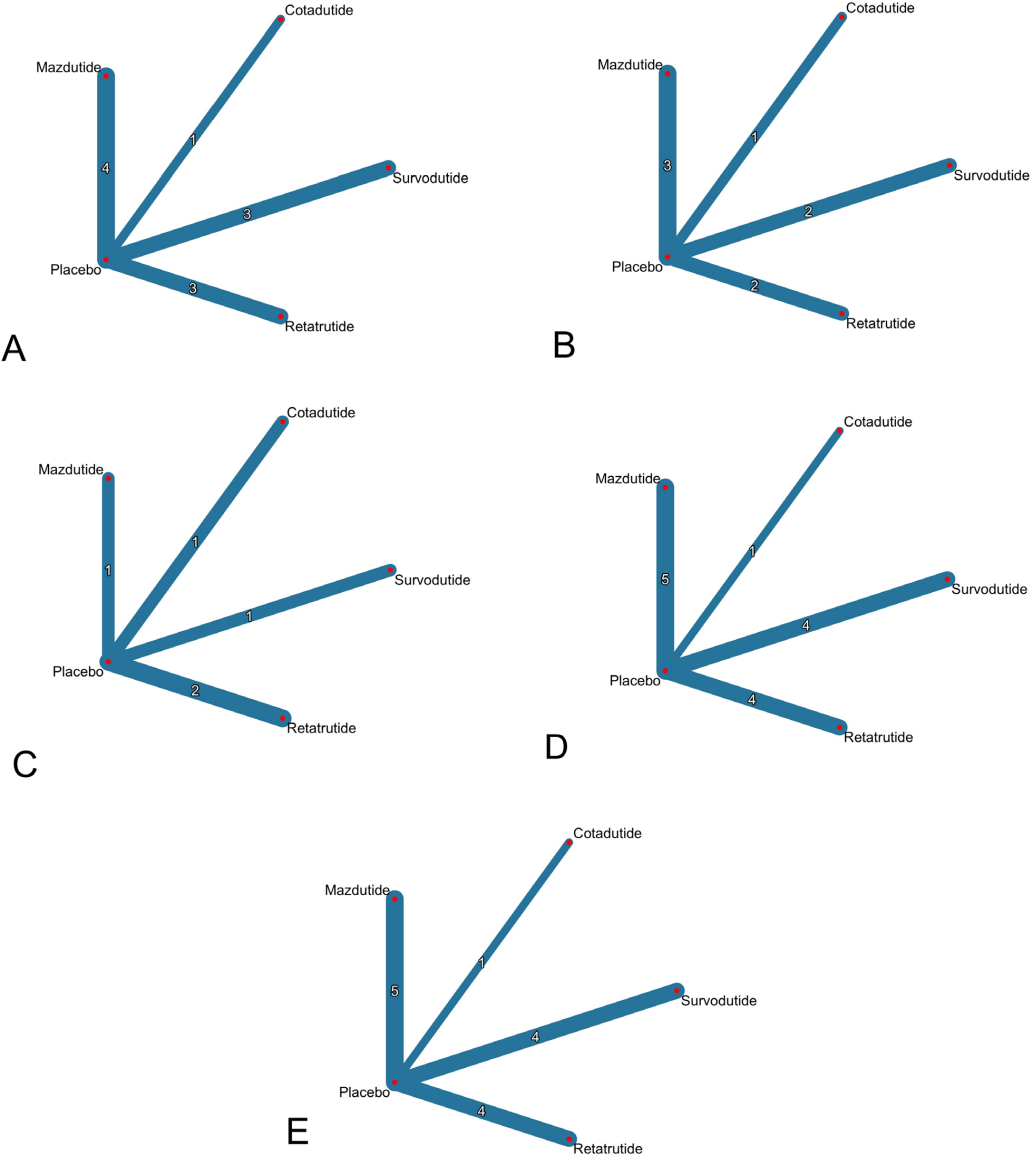
Meta‐analysis networks for (A) absolute change in weight (kg), (B) change in HbA1c, (C) percent change in body weight, (D) adverse events, and (E) treatment discontinuation rate due to adverse events. Each circle indicates a treatment node. Lines connecting two nodes represent direct comparisons between two treatments; the thickness of the lines is proportional to the number of trials directly comparing the two connected treatments.

A total of 14 randomised controlled trials involving 3102 participants were included. All studies investigated the efficacy and tolerability of retatrutide, survodutide, cotadutide, or mazdutide in patients with diabetes, overweight and/or obesity. We identified 4 trials investigating survodutide, 4 trials for retatrutide, 5 trials for mazdutide, and 1 trial for cotadutide. The network for these included studies for evaluating weight loss, HbA1c, weight percent, adverse events, and treatment discontinuation rates was shown in Figure 2A–E and Supplement 8.1 to 8.5, respectively, and the characteristics of studies and patients' baseline features are presented in Supplements 2 and 3.

### 3.2 Efficacy Outcome

#### 3.2.1 Absolute Change in Weight (Kg)

**FIGURE 3:**
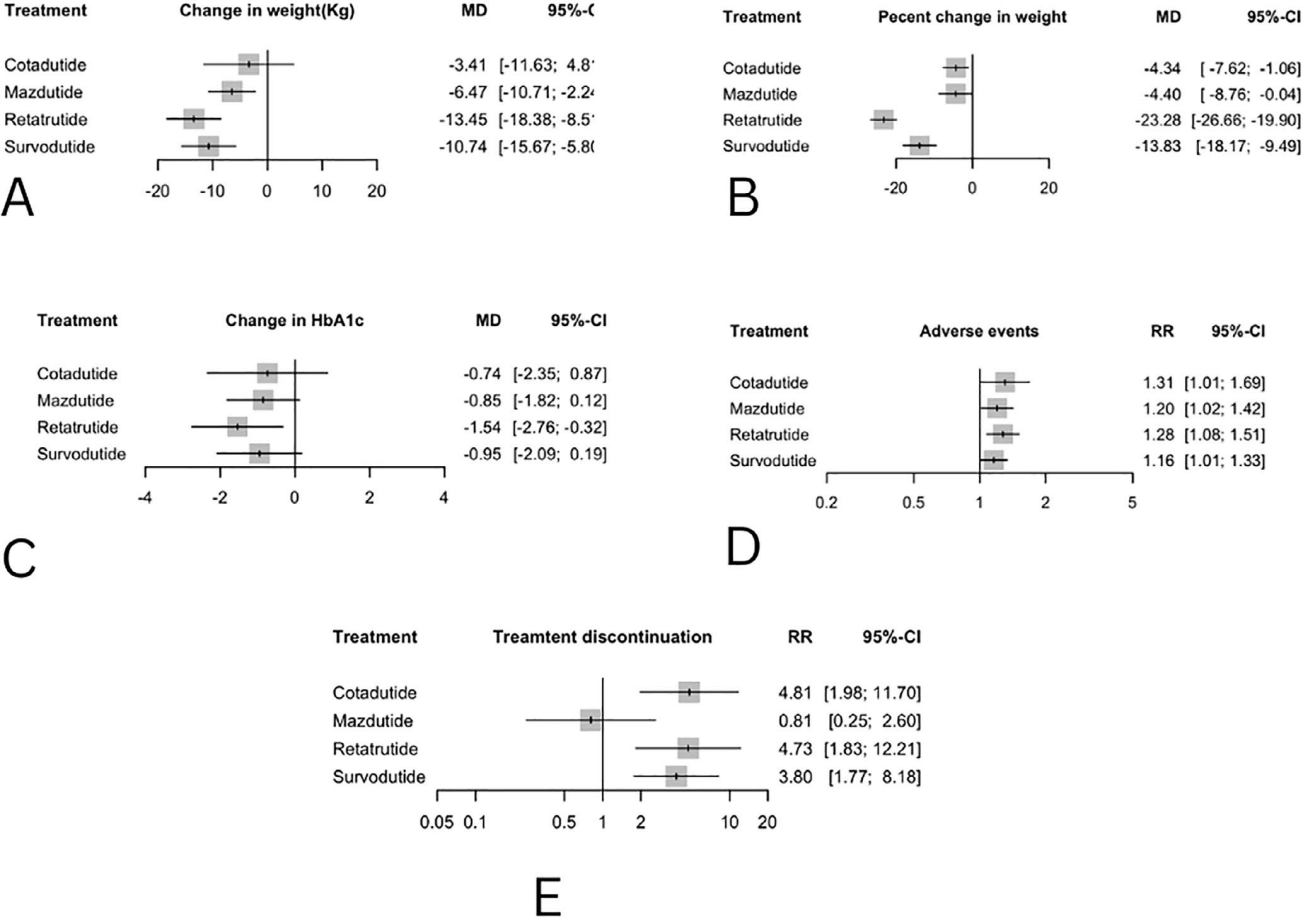
Network meta‐analysis results for (A) change from baseline in body weight (kg), (B) percent change in body weight, (C) change from baseline in HbA1c (%), (D) incidence of adverse events, and (E) treatment discontinuation due to adverse events compared with placebo. Effect sizes are presented as mean difference (MD) and 95% confidence intervals (CI). For (A–C), treatments are presented according to their effect estimates compared with placebo. For (D and E), treatments are presented according to their effect estimate compared with placebo. Effect sizes are presented as risk ratio (RR) and 95% confidence intervals (CI).

A total of 11 RCTs reported absolute weight change. Direct pairwise meta‐analyses showed that retratrutide, followed by survodutide, were associated with a significant reduction in body weight compared with placebo, presented in Supplement 6.1. These findings were consistent with the network meta‐analysis estimates presented in Figure 3A, and Supplement 7.1. Retatrutide demonstrated the most substantial reduction in body weight with a mean difference (MD) of −13.45 kg (95% Confidence Interval (CI): −18.38 to −8.51; *p* < 0.00), followed by survodutide (MD −10.74 kg; 95% CI: −15.68 to −5.80; p < 0.00) and mazdutide (MD −6.47 kg; 95% CI: −10.71 to −2.24; *p* = 0.002). Cotadutide resulted in the least weight reduction (MD −3.41 kg; 95% CI: −11.63 to 4.81; *p* = 0.41).

The estimated treatment ranking based on P‐scores indicated that retatrutide had the highest likelihood of being the most effective treatment (*P*‐score = 0.93), followed by survodutide (0.77), mazdutide (0.51), and lastly cotadutide (0.30).

Substantial heterogeneity in the primary outcome was observed (*I* ^2^ = 94.4%), indicating notable variability among the included studies. However, leave‐one‐out sensitivity analysis estimates remained stable when each study was excluded individually, supporting the robustness of the findings. Funnel plots were examined to provide additional assurance of consistency across studies, see Supplement 4.1.

Meta‐regression against age, male, BMI, and duration did not reveal statistically significant differences across treatment groups for any of the evaluated characteristics, indicating that the transitivity assumption was reasonably upheld. Certainty of evidence according to the CINeMA framework was rated as low due to concerns of heterogeneity and imprecision, see supplement 5.1.

#### 3.2.2 Percent Change in Weight

A total of 5 studies were included in the main analysis for the change in weight percent. Direct pairwise meta‐analysis against placebo is presented in Supplement 6.2, showing that retatrutide was associated with the greatest magnitude of weight loss (MD −23.28, 95% CI [−26.66; −19.90]) followed by survodutide (MD −13.83, 95% CI [−18.17; −9.48]), mazdutide, and cotadutide, respectively. These findings were consistent with the network meta‐analysis estimates, which are presented in Figure 3B, and Supplement 7.2.

Based on P‐scores from the network meta‐analysis, retatrutide ranked highest for weight reduction (P‐score 0.99), followed by survodutide (0.74), cotadutide (0.37), and mazdutide (0.37).

Heterogeneity was low to moderate (*I* ^2^ at 34.4%; *p* = 0.217), indicating limited variability among studies. Sensitivity analyses confirmed the robustness of the findings, with the effects on percent weight change remaining stable across all analyses. Univariable meta‐regression suggested that age may partially explain variability in percent weight change between studies, while sex, baseline BMI, and study duration did not. Due to the limited number of studies, a multivariable analysis could not be performed. The CINeMA assessment resulted in low certainty of evidence due to within‐study bias, reporting bias, imprecision, and heterogeneity in the included studies, see Supplement 4.2 and 5.2.

#### 3.2.3 Absolute Change in Haemoglobin A1c

A total of 8 studies were included in the main analysis for the change in HbA1c. Direct pairwise meta‐analyses showed that retatrutide demonstrates the highest levels of HbA1C reduction from placebo compared to other GRAs (MD −1.54 [−2.76; −0.32]; *p* = 0.01), followed by survodutide (MD −0.95 [−2.08; 0.18]; *p* = 0.1), mazdutide (MD −0.84 [−1.82; 0.12]; *p* = 0.08), and lastly, cotadutide showed (MD −0.74 [−2.34; 0.86]; *p* = 0.36), see Supplement 6.3. These findings were supported by network meta‐analysis results, which are presented in Figure 3C, and Supplement 7.3. Furthermore, among all the interventions, retatrutide ranked highest (*P*‐score = 0.83), followed by survodutide, mazdutide, and cotadutide, respectively.

Substantial heterogeneity was observed across studies (*I* ^2^ = 95.5%; *p* = 0.002), suggesting that the combined influence of covariates (sex, age, BMI, and intervention duration) accounts for a significant portion of the variability in HbA1c outcomes across studies. Sensitivity analyses showed that the HbA1c estimates stayed generally stable under different modelling assumptions. Excluding studies with a high risk of bias or small sample sizes did not significantly change the results. This indicates that the pooled estimates are robust. Meta‐regression analyses showed that age was significantly associated with the HbA1c in univariable analysis; however, this effect did not persist in multivariable models.

The certainty of evidence was rated as low, mainly due to concerns related to within‐study bias, reporting bias, imprecision, and heterogeneity across included trials (see Supplement 4.3 and 5.3).

While efficacy outcomes favoured retatrutide and survodutide across weight and glycaemic measures, interpretation of these findings should consider tolerability profiles, as greater metabolic benefits may be accompanied by higher rates of gastrointestinal adverse events and treatment discontinuation.

### 3.3 Safety Outcomes

#### 3.3.1 Discontinuation Rate due to Adverse Events

Fourteen studies were included for this outcome. Direct pairwise meta‐analyses showed that mazdutide demonstrates the lowest treatment discontinuation rate (RR = −0.21, [−1.38; 0.95]; *p* = 0.72), followed by survodutide with a moderate rate (RR =1.33, [0.56; 2.10]; *p* = 0.00), while retatrutide (RR =1.55, [0.60; 2.50]; *p* = 0.00) and cotadutide (RR = 1.57, [0.68; 2.45]; *p* = 0.00) exhibit the highest discontinuation rates due to adverse events. Network meta‐analysis results are presented in Figure 3E and Supplement 7.5, which were consistent with the pairwise findings, confirming the relative tolerability of the treatments, see Supplement 6.5.

Based on P‐scores, mazdutide ranked as the most favourable treatment for tolerability (0.90), followed by survodutide, while retatrutide and cotadutide had the highest risk of discontinuation due to adverse events.

Notably, the overall heterogeneity across all studies was *I* ^2^ = 0%, indicating consistency among the results. Leave‐one‐out sensitivity analysis estimates remained stable when each study was excluded individually, supporting the robustness of the findings. Funnel plots did not detect any degree of publication bias for this outcome; see Supplement 4.5. The certainty of evidence for treatment discontinuation was rated as low; see Supplement 5.5.

#### 3.3.2 Adverse Events

14 studies were included in the analysis of adverse events. Notably, according to the pairwise meta‐analyses, the four GRAs in this study did not show statistically significant differences in the incidence of adverse events. However, each showed a statistically significant difference compared to placebo: cotadutide (RR = 0.26, 95% CI: [0.011; 0.52]; *p* = 0.04), mazdutide (RR = 0.18, [0.018; 0.34]; *p* = 0.02), retatrutide (RR = 0.24, [0.074; 0.41]; *p* = 0.00), and survodutide (RR = 1.14, [0.006; 0.28]; *p* = 0.04), see Supplement 6.4. Network meta‐analysis results presented in Figure 3D and Supplement 7.4, were consistent with the pairwise findings, as survodutide demonstrated the most favourable safety profile, followed by mazdutide, while retatrutide and cotadutide consistently showed the highest risk of adverse events relative to placebo.

Heterogeneity across studies was low to moderate (*I* ^2^ = 39.3%). Leave‐one‐out analyses showed that no individual study altered the overall results. Leave‐one‐out sensitivity analyses showed stable estimates after sequential exclusion of individual studies, supporting the robustness of the adverse events findings. The certainty of evidence remained low due to concerns of study quality and imprecision, see Supplement 5.4.

We analysed several common side effects of GRAs, the most reported across all studies are gastrointestinal side effects (nausea, vomiting, diarrhoea, and dyspepsia), see Supplement 4.4. However, the absolute incidence of adverse events could not be pooled due to inconsistent reporting across trials, limiting quantitative comparison.

#### 3.3.3 Vomiting

All glucagon receptor agonists showed statistically significant higher risks of vomiting than placebo. Cotadutide demonstrated an RR of (1.34), Mazdutide (1.44), Retatrutide (1.43), and Survodutide (1.28), with those consistent with network meta‐analysis results shown in Figure 5A. Survodutide demonstrated the best tolerability profile (*P*‐score = 0.47), followed by Cotadutide (0.40), Retatrutide (0.31), and Mazdutide (0.31). This suggests that Mazdutide had the least favourable results regarding vomiting. Heterogeneity was negligible (*I* ^2^ = 0%)., and sensitivity analysis indicates robustness, See Supplement 4.6.

#### 3.3.4 Diarrhoea

Direct pairwise meta‐analyses demonstrate that Mazdutide (RR 0.77), Retatrutide (0.72), and Survodutide (0.85) show statistically significant increased risk of diarrhoea compared with placebo. However, Cotadutide (0.2) does not show a significant difference. These findings are consistent with network meta‐analysis results shown in Figure 5B. The ranking of treatments indicated that Cotadutide had the optimal diarrhoea profile (P‐score = 0.71), followed by Survodutide (0.38), Retatrutide (0.26), and Mazdutide (0.21), implying that Mazdutide presented the least favourable diarrhoea profile. The level of heterogeneity stayed minimal (*I* ^2^ = 0%). Sensitivity analyses showed stable estimates, supporting the robustness. See Supplement 4.7.

#### 3.3.5 Nausea

Direct pairwise meta‐analyses demonstrated that Cotadutide (RR 1.34), Mazdutide (RR 1.32), Retatrutide (RR 1.43), and Survodutide (RR 1.28) were all associated with a statistically significant increase in nausea compared with placebo. Network meta‐analysis results shown in Figure 5C. Treatment ranking identified Survodutide as having the most favourable profile (*P*‐score = 0.44), followed by Mazdutide (0.39), Cotadutide (0.37), and Retatrutide (0.28), suggesting that Retatrutide had the least favourable profile for Nausea. Heterogeneity was minimal (*I* ^2^ = 0%). Sensitivity analyses showed stable estimates, supporting the robustness, See Supplement 4.8.

#### 3.3.6 Dyspepsia

For dyspepsia, all agents demonstrated increased risks compared with placebo. Cotadutide had an RR of (1.34), Mazdutide (1.32), Retatrutide (1.43), and Survodutide (1.28). Network meta‐analysis results shown in Figure 5D. Survodutide ranked most favourably (P‐score = 0.44), followed by Mazdutide (0.39), Cotadutide (0.37), and lastly Retatrutide (0.28). Heterogeneity was negligible (I 2 = 0%). Sensitivity analyses showed stable estimates, supporting the robustness. See Supplement 4.9.

## 4 Discussion

In NMA of 14 randomised controlled trials [9, 10, 11, 12, 13, 14, 15, 16, 17, 18, 19, 20, 21, 22], the effectiveness and safety profile of the latest developed GRAs was evaluated in patients with T2DM and/or overweight and obesity. These populations were studied together because excess body weight and dysregulated glucose metabolism are closely interrelated pathophysiological processes. Weight reduction is a common therapeutic target in both obesity and T2DM. Accordingly, these agents are commonly used across overlapping clinical indications as weight loss is consistently associated with concurrent improvements in glycaemic control, insulin sensitivity, and overall cardiometabolic risk.

Retatrutide and Survodutide were associated with the largest reductions in body weight and HbA1c. Retatrutide achieved the mean absolute weight loss of −13.45 kg, followed by Survodutide, Mazdutide, and Cotadutide. In terms of HbA1c reduction, Retatrutide is also the highest (−1.54%), followed by Survodutide, Mazdutide, and Cotadutide. Although Retatrutide and Survodutide generally ranked highest, overlapping confidence intervals suggest that some differences between treatments should be interpreted with caution.

Regarding safety outcomes, the lowest rates of discontinuation due to adverse events were recorded for Mazdutide, followed by Survodutide, Retatrutide, and Cotadutide.

Balancing these efficacy findings with tolerability is critical as higher metabolic benefits may be associated with increased risk of gastrointestinal adverse events and treatment discontinuation.

These findings underline Retatrutide and Survodutide as the most promising candidates among the emerging GRAs, offering a compelling combination of efficacy and tolerability for obesity treatment. Retatrutide appears especially beneficial for individuals with T2DM, whereas Survodutide did not show a significant impact on glycaemic control in our analysis, indicating the need for further research. Both agents achieved substantial reductions in body weight and HbA1c. These results exceed generally accepted thresholds for meaningful metabolic improvements (≥ 5% reduction in body weight and ≥ 0.5% reduction in HbA1c) and establish the high therapeutic value of glucagon receptor‐based agonists in addressing the dual challenges of obesity and T2DM. Given the growing demand for treatments that improve both parameters, the efficacy of these agents is noteworthy. However, their long‐term use may be influenced by tolerability considerations [29].

In our study of GRAs, tolerability varied among the agents. Mazdutide had higher risks of vomiting and diarrhoea, showing the least favourable gastrointestinal profile in these areas. Retatrutide also had more gastrointestinal side effects, especially nausea and dyspepsia. In contrast, Survodutide showed the best tolerability across all gastrointestinal outcomes. Cotadutide had an intermediate profile, with lower risks of diarrhoea and vomiting compared to Mazdutide and Retatrutide. On the other hand, recent evidence suggests that Retatrutide is generally well tolerated, showing no statistically significant difference in tolerability compared with approved marketed GLP‐1 only agonist while offering superior efficacy in both glycaemic and weight reduction outcomes [30]. These findings highlight that triple agonists like Retatrutide may provide optimal metabolic benefits with acceptable tolerability, reflecting an evolving therapeutic balance between efficacy and side effects. Overall, these findings indicate a shift in treatment paradigms, where the balance between efficacy and tolerability will guide clinical decision‐making. As evidence accumulates, these compounds are likely to shape future guidelines and redefine therapeutic strategies in metabolic care.

GRAs offer a unique and promising approach in metabolic therapy. They work primarily by increasing energy expenditure and promoting weight loss independently of GLP‐1‐mediated appetite suppression. This is achieved by enhancing the metabolic rate through the stimulation of hepatic glucose production and activating thermogenic pathways in brown adipose tissue [31]. Agents such as Survodutide and Mazdutide, which co‐activate GLP‐1 and glucagon receptors, harness this pathway to achieve substantial weight reduction, with glucagon‐driven thermogenesis and energy expenditure being central to their efficacy [23, 32, 33]. Among these, Mazdutide shows a relatively less favorable tolerability profile than Survodutide, likely due to differences in receptor activation balance.

Retatrutide, a triple agonist targeting GLP‐1, GIP, and glucagon receptors, builds upon this glucagon‐centred effect by adding GIP activity, which enhances insulin sensitivity and fat metabolism. This combination leads to superior HbA1c and weight reductions, representing the highest efficacy observed to date [30]. However, the gastrointestinal side effects seen with Retatrutide are mainly due to GLP‐1 receptor activation. These effects seem to be dose‐dependent. This highlights the need to balance effectiveness and tolerability. Dual agonists like Cotadutide, while similar to Mazdutide and Survodutide, have a receptor profile skewed towards liver targeting and may produce less central appetite suppression, which could explain their more modest metabolic effects [34]. Interindividual variations in receptor sensitivity, metabolic rate, and incretin resistance, particularly in T2DM patients, may further influence outcomes.

Alongside glucagon‐mediated increases in energy expenditure, the GLP‐1 part of dual and triple agonists offers other important effects. Research indicates that GLP‐1 signalling affects several body systems, including gastrointestinal, cardiovascular, renal, and neuroendocrine pathways. This supports appetite control, blood sugar management, and overall metabolic balance. These effects across multiple systems help clarify the improved clinical results seen with drugs that combine GLP‐1 and glucagon receptor activity. At the same time, the gastrointestinal effects related to GLP‐1 show the need to balance effectiveness with tolerability when assessing these treatments in real‐world settings [35, 36, 37, 38].

**FIGURE 4:**
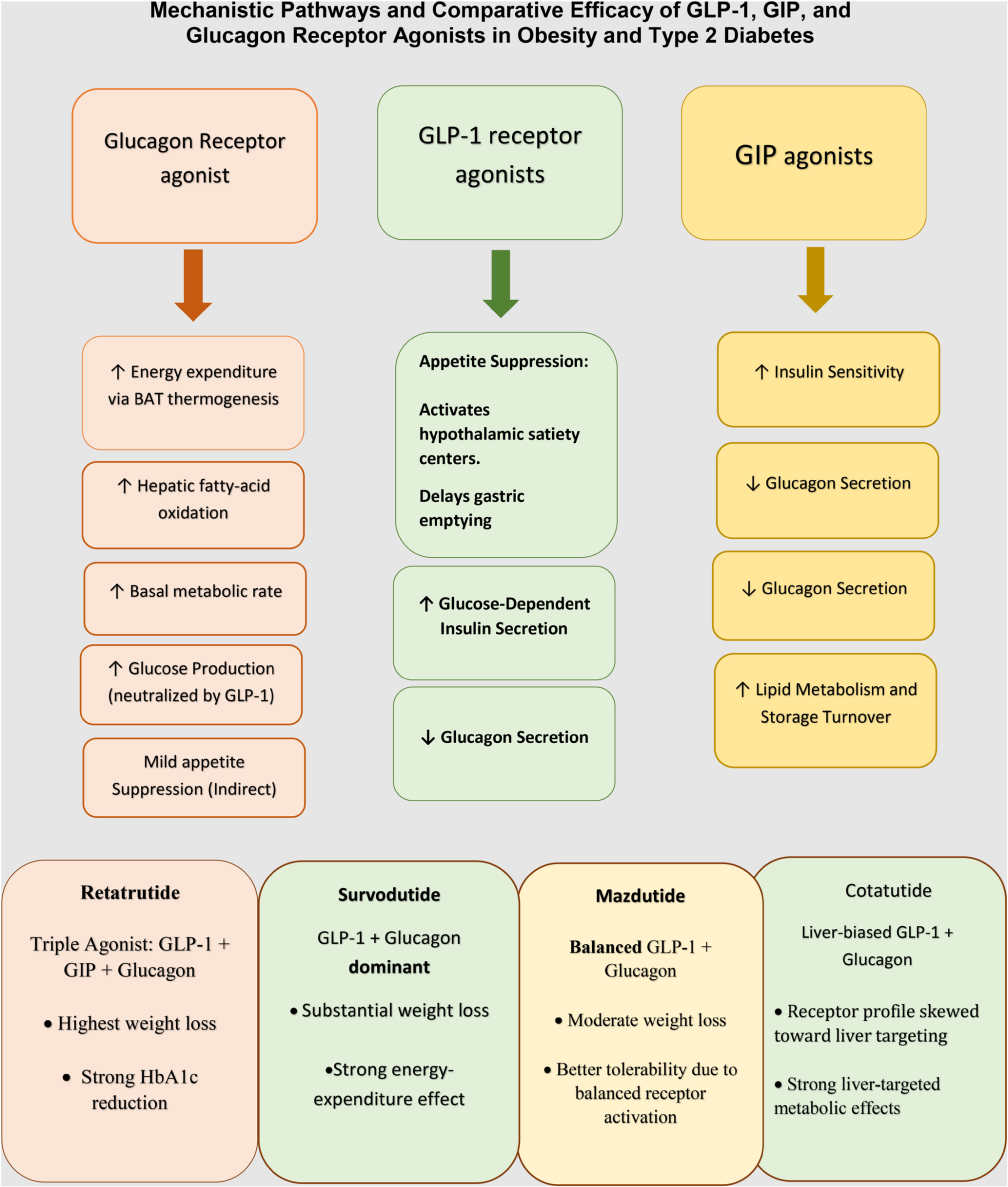
Mechanistic Pathways and Comparative Efficacy of GLP‐1, GIP, and Glucagon Receptor Agonists in Obesity and Type 2 Diabetes.

These mechanistic classifications closely match the comparative outcomes observed in our network meta‐analysis. Among all the agents, Retatrutide achieved the highest level of weight loss, which is consistent with the additive metabolic effects from the concurrent activation of GLP‐1, GIP, and glucagon receptors. Survodutide and Mazdutide demonstrated the next highest levels of efficacy, which correspond with their dual action as GLP‐1/glucagon agonists and the glucagon‐driven boosts in energy expenditure. However, Survodutide showed better efficacy due to its more glucagon‐dominant dual‐agonist profile, which produces stronger thermogenic activation and greater increases in basal metabolic rate. In contrast, Mazdutide engages GLP‐1 and glucagon receptors in a more balanced manner, resulting in moderate thermogenesis with improved tolerability and more stable glycemic control. Cotadutide produced more modest effects in our analysis, which parallels its comparatively lower central appetite suppression and liver‐skewed receptor activity. This alignment from mechanistic insights to clinical application supports the biological validity of our findings; these relationships are visually summarised in Figure 4.

**FIGURE 5:**
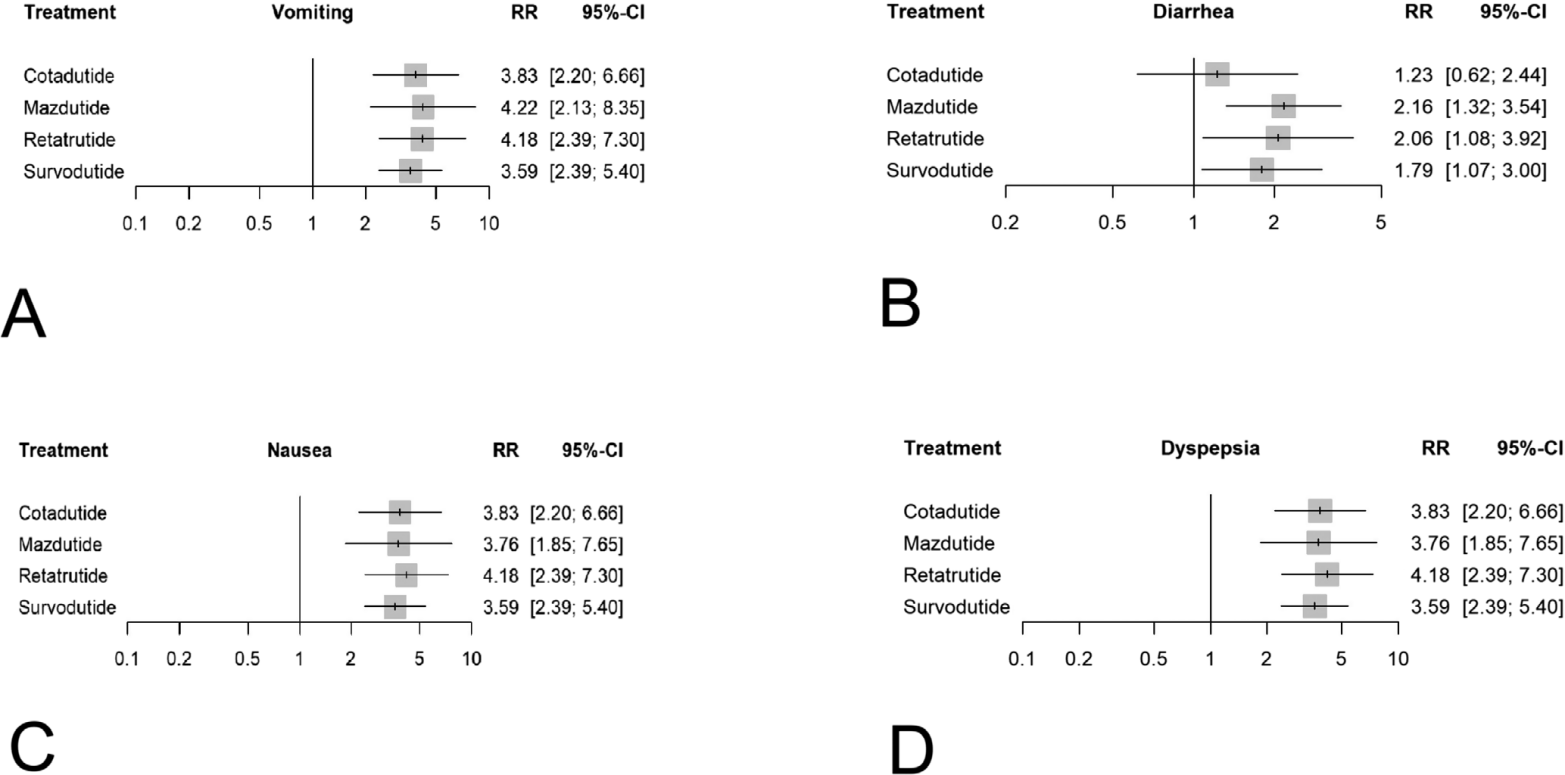
Meta‐analysis networks results for (A) vomiting, (B) diarrhea, (C) nausea, (D) and dyspepsia. Treatments are presented according to their effect estimate compared with placebo. Effect sizes are presented as risk ratio (RR) and 95% confidence intervals (CI).

Our analysis aligns with prior meta‐analyses and systematic GRAs reviews. Retatrutide's robust effects on weight (MD −13.44 kg) and HbA1c (MD −1.54%) are consistent with findings reported by Tewari et al. and Pasqualotto et al., reinforcing its role as a dual‐action agent. However, it also demonstrates the highest rate of discontinuation due to adverse events [39, 40, 41]. Survodutide demonstrated significant weight reduction in our review, which corresponds with the outcomes described by Wan et al., adding further support to its emerging evidence base [42]. Cotadutide's modest results, in addition to its high rate of treatment discontinuation due to adverse events, mirror those from Ali et al. [39], suggesting a more limited role in practice. Mazdutide, which showed moderate but consistent improvements, aligns with reports from Nalisa et al., supporting its potential for balanced efficacy and tolerability [43]. Overall, Retatrutide ranked highest in efficacy, followed by Survodutide, with Mazdutide offering a potentially more tolerable option and Cotadutide playing a more limited place in therapy.

This network meta‐analysis offers several strengths. Notably, it is the first to include Survodutide, adding timely and original evidence to the literature. By systematically searching both PubMed, Embase, Cochrane, and Scopus and including only randomised controlled trials with at least 12 weeks of follow‐up, we ensured methodological rigour and internal validity. Our frequentist network meta‐analytic approach enabled a robust comparison of treatments, incorporating both direct and indirect evidence to rank interventions. Sensitivity analyses, including leave‐one‐out tests, exclusion of high‐risk‐of‐bias or small‐sample studies, and use of alternative model specifications, confirmed that our findings were robust. The observed effects on weight loss, HbA1c reduction, and safety outcomes remained stable, indicating that no single study or analytic choice disproportionately influenced the results. We also employed the CINeMA framework and performed a structured transitivity assessment based on baseline trial characteristics to enhance interpretability.

Transitivity, a key assumption in network meta‐analysis, was carefully assessed. We evaluated baseline characteristics like age, BMI, diabetes duration, and treatment history to ensure that comparisons between agents were clinically reasonable. While some variability existed, no major violations were detected that would undermine the validity of indirect comparisons.

However, there are limitations. Considerable heterogeneity across primary and secondary outcomes was observed, reflecting variations in study design, populations, and particularly the wide range of dosing regimens used across trials. While all the included trials met the minimum 12‐week requirement, most had follow‐up periods ranging from 16 to 46 weeks. This makes it hard to conclude about the long‐term durability of weight loss and safety. Our primary analysis focused on the highest available doses, which could overestimate treatment effects and underestimate adverse events. Also, the evidence mainly came from early‐phase trials (Phase 1b and Phase 2), and the network was predominantly built around placebo‐controlled trials, with no direct head‐to‐head comparisons among active agents, increasing reliance on indirect estimates. Interpreting the results necessitates an examination of these variables collectively.

## 5 Implications for Practice and Future Research

This work reinforces the therapeutic promise of newer glucagon receptor agonists in obesity and diabetes care. However, several gaps remain. Future research should prioritise direct head‐to‐head trials, especially between Retatrutide and Survodutide, to clarify their relative advantages. Long‐term studies examining durability of response, cardiovascular outcomes, and safety beyond 36 weeks are essential. Additionally, trials should include diverse populations, such as older adults, patients with advanced disease, and those intolerant to current treatments, to improve generalizability. Importantly, consistent evaluation of patient‐centred outcomes, including adherence and quality of life, will be critical to guide personalised treatment strategies.

## 6 Conclusion

Among the new dual and triple glucagon receptor agonists, Retatrutide and Survodutide showed the best efficacy in glycaemic control and weight loss in this network meta‐analysis of RCTs. In terms of therapeutic efficacy, Retatrutide was the most effective, while Survodutide was a strong contender. While Cotadutide demonstrated the lowest efficacy and the highest rates of dropout, Mazdutide demonstrated intermediate effectiveness with tolerability. To support these findings and direct future practice, more head‐to‐head studies and long‐term safety data are required.

## Author Contributions

**Hazem Ayesh:** conceptualization, methodology, software, supervision, writing – review and editing, validation, visualization, investigation, formal analysis, data curation, resources. **Doha Jaber:** investigation, writing – review and editing, writing – original draft, visualization, methodology. **Ayah Abulehia:** conceptualization, investigation, writing – original draft, methodology, visualization, writing – review and editing, project administration, data curation, validation, software, resources.

## Funding

The authors have nothing to report.

## Conflicts of Interest

The authors declare no conflicts of interest.

## Supporting information


**Data S1:** Supporting Information.

## Data Availability

The data that support the findings of this study are available in Pubmed at https://pubmed.ncbi.nlm.nih.gov/. These data were derived from the following resources available in the public domain: Pubmed, https://pubmed.ncbi.nlm.nih.gov/‐ Scopus, https://www.Elsevier.com/products/Scopus.
