# Supplementary material for: Comparative Efficacy and Safety of Glucagon Receptor Agonists on Metabolic Outcomes: A Network Meta‐Analysis of Randomised Controlled Trials
Source: Endocrinol Diabetes Metab. 2026 Mar 5;9(2):e70187. doi: 10.1002/edm2.70187 (PMC12963462; doi:10.1002/edm2.70187)
Supplement: Supplementary file 1 — Data S1: Supporting Information. [file EDM2-9-e70187-s001.docx]

**Supplementary Material**

Contents

[Supplement 1. Search strategy 5](#_Toc216388323)

[Supplement 2: Characteristics of included studies 6](#_Toc216388324)

[Supplement 3: baseline characteristics of included studies 9](#_Toc216388325)

[Supplement 4: Publication bias -funnel plot- for each outcome 11](#_Toc216388326)

[*S4.1: Publication bias -funnel plot- for absolute weight* 11](#_Toc216388327)

[*S4.2: Publication bias -funnel plot- for percent weight* 12](#_Toc216388328)

[*S4.3: Publication bias -funnel plot- for HbA1c* 13](#_Toc216388329)

[*S4.4: Publication bias -funnel plot- for adverse events* 14](#_Toc216388330)

[*S4.5: Publication bias -funnel plot- for discontinuation of treatment due to adverse events:* 15](#_Toc216388331)

[*S4.6: Publication bias -funnel plot- for vomiting:* 15](#_Toc216388332)

[*S4.7: Publication bias -funnel plot- for Diarrhea:* 16](#_Toc216388333)

[*S4.8: Publication bias -funnel plot- for Nausea:* 17](#_Toc216388335)

[*S4.9: Publication bias -funnel plot- for Dyspepsia:*  17](#_Toc216388336)

[Supplement 5: Certainty of evidence for each outcome 18](#_Toc216388337)

[*S5.1: Certainty of evidence for absolute weight* 18](#_Toc216388338)

[*S5.2: Certainty of evidence for percent weight* 19](#_Toc216388339)

[*S5.3: Certainty of evidence for HbA1c* 20](#_Toc216388340)

[*S5.4: Certainty of evidence for adverse events* 21](#_Toc216388341)

[*S5.5: Certainty of evidence for treatment discontinuation due to adverse events* 22](#_Toc216388342)

[Supplement 6. Network meta-analysis results (league) 23](#_Toc216388343)

[6.1. Network meta-analysis results for change in absolute weight (kg) 23](#_Toc216388344)

[6.2. Network meta-analysis results for change in weight percent 23](#_Toc216388345)

[6.3. Network meta-analysis results for Change from baseline in HbA1c 23](#_Toc216388346)

[6.4. Network meta-analysis results for adverse events: 24](#_Toc216388347)

[6.5. Network meta-analysis results for treatment discontinuation due to adverse events 24](#_Toc216388348)

[Supplement 7: Network meta-analysis results for each outcome 25](#_Toc216388349)

[S7.1: Network meta-analysis results for absolute weight loss 25](#_Toc216388350)

[S7.2: Network meta-analysis results for Percent weight changes 25](#_Toc216388351)

[26](#_Toc216388352)

[S7.3: Network meta-analysis results for HbA1c 26](#_Toc216388353)

[26](#_Toc216388354)

[S7.4: Network meta-analysis results for adverse events 26](#_Toc216388355)

[27](#_Toc216388356)

[S7.5: Network meta-analysis results for treatment discontinuation due to adverse events: 27](#_Toc216388357)

[Supplement 8: Network geometry for each outcome 28](#_Toc216388358)

[*S8.1 Network geometry for absolute weight:* 28](#_Toc216388359)

[*S8.2 Network geometry for percent weight:* 29](#_Toc216388360)

[29](#_Toc216388361)

[*S8.3 Network geometry for HbA1c* 30](#_Toc216388362)

[*S8.4 Network geometry for adverse events* 30](#_Toc216388363)

[*S8.5 Network geometry for treatment discontinuation due to adverse events* 31](#_Toc216388364)

[Supplement 9. Risk of bias assessment of included trials for each outcome 32](#_Toc216388365)

[Supplement 10: PRISMA checklist 34](#_Toc216388366)

# Supplement 1. Search strategy

From inception to February 15^th^, 2025:

**PubMed:**("Survodutide" OR "BI 456906" OR "Retatrutide" OR "LY3437943" OR "Cotadutide" OR "MEDI 0382" OR "Mazdutide" OR “IBI362” OR “LY3305677” OR "Dual GLP-1 glucagon agonist" OR "GLP-1 glucagon co-agonist") AND ("Obesity"[Mesh] OR "Type 2 Diabetes Mellitus"[Mesh] OR "Diabetes Mellitus"[Mesh]) AND "Clinical Trial"[Publication Type]

**Scopus:**

("Survodutide" OR "BI 456906" OR "Retatrutide" OR "LY3437943" OR "Cotadutide" OR "MEDI 0382" OR "Mazdutide" OR "IBI362" OR "LY3305677" OR "Dual GLP-1 glucagon agonist" OR "GLP-1 glucagon co-agonist") AND ("Obesity" OR "Type 2 Diabetes Mellitus" OR Diabetes Mellitus") AND "Clinical Trial"

**Cochrane:**("Survodutide" OR "BI 456906" OR "Retatrutide" OR "LY3437943" OR "Cotadutide" OR "MEDI 0382" OR "Mazdutide" OR "IBI362" OR "LY3305677" OR

"Dual GLP-1 glucagon agonist" OR "GLP-1 glucagon co-agonist") AND ("obesity" OR "type 2 diabetes" OR "diabetes mellitus")

AND ("clinical trial") **Embase:**

('survodutide'/exp OR 'survodutide' OR 'bi 456906' OR 'retatrutide'/exp OR 'retatrutide' OR 'ly3437943' OR 'cotadutide'/exp OR 'cotadutide' OR 'medi 0382' OR 'mazdutide'/exp OR 'mazdutide' OR 'ibi362' OR 'ly3305677' OR 'dual glp-1 glucagon agonist' OR 'glp-1 glucagon co-agonist') AND ('obesity'/exp OR 'obesity' OR 'type 2 diabetes mellitus'/exp OR 'type 2 diabetes' OR 'diabetes mellitus'/exp OR 'diabetes mellitus') AND ('clinical trial'/exp OR 'clinical trial')

| Supplement 2: Characteristics of included studies  \| **Population** \| **Duration** \| **Comperator** \| **Dose** \| **Intervention** \| **Registration** \| **Year** \| **Design** \| **Study** \| \| --- \| --- \| --- \| --- \| --- \| --- \| --- \| --- \| --- \| \| adults who had a BMI of 30 or higher or who had a BMI of 27 to less than 30 plus at least one weight-related condition \| 48 \| Placebo \| 1 mg, 4 mg (ID 2 mg or ID 4 mg), 8 mg (ID 2 mg or ID 4 mg), and 12 mg (ID 2 mg) \| Retatrutide \| NCT04881760 \| 2023 \| Phase2 \| Jastreboff,2023 \| \| 18–75 years, had been diagnosed with T2DM for ≥6 months, had an HbA1c value of (7.0–10.0%) and a BMI of 25–50 kg/m2 at screening and had been treated with a stable dose of metformin of ≥1000 mg/day (immediate or extended release) for ≥3 months before screening \| 16 \| Placebo \| 0.3 mg, 0.9 mg, 1.8 mg, and 2.7 mg qw, and 1.2 mg and 1.8 mg biw \| Survodutide \| NCT04153929 \| 2023 \| Phase2 \| Blüher,2023 \| \| Adults (aged 18–70 years) with a BMI of 27–40 kg/m2 and stable body weight (≤5% change within 3 months prior to screening) of 70 kg or higher (females) or 80 kg or higher (males). not exposed to any GLP-1RAs within 12 months prior to screening \| 16 \| Placebo \| 16.39 mg, 38.24 mg, 42.60 mg \| Survodutide \| NCT03175211 \| 2022 \| Phase1 \| Jungnik,2022 \| \| Japanese men aged 20 to 45 years, with a BMI of 23 to 40 kg/m2, stable (≤5% change within 3 months) bodyweight of ≥65 kg and glycated haemoglobin (HbA1c) levels of <6.5%.not exposed to any GLP-1RAs within 12 months prior to screening \| 16 \| Placebo \| 1.8 mg,4.8 mg, 2.4 mg \| Survodutide \| NCT04384081 \| 2023 \| Phase1 \| Yazawa,2023 \| \| Patients (aged 18–75) who had been diagnosed with T2D for at least six months that was inadequately controlled with diet and exercise alone or with stable metformin therapy (glycated haemoglobin A1c [HbA1c] 7.5–11.0%, both inclusive) and a body-mass index (BMI) of 20–35 kg/m2 \| 12 \| Placebo \| 3 mg, 4.5 mg, 6 mg \| Mazdutide \| NCT04466904 \| 2022 \| Phase1b \| Jiang,2022 \| \| Adults with overweight (24 kg/m2) accompanied by hyperphagia and/or at least one comorbidity (pre- diabetes, hypertension, dyslipidemia, fatty liver, weight-bearing  arthralgia or dyspnea, obstructive sleep apnea syndrome caused by obesity) or obesity (BMI 28 kg/m2 ) and with less than 5% weight loss by diet and exercise 12 weeks or longer prior to screening were eligible for this study. The key exclusion criteria were concurrent or previous use of GLP-1 receptor agonists, use of weight-loss or anti-obesity agents three months prior to screening." \| 12 \| Placebo \| 3 mg, 4.5 mg, 6 mg \| Mazdutide \| NCT04440345 \| 2021 \| Phase1b \| Ji,2021 \| \| Adults (aged 18-75 years, both inclusive) with over-weight ([BMI] ≥24 kg/m2) accompanied by hyperphagia and/or at least one obesity-relatedcomorbidity (pre-diabetes, hypertension, dyslipidaemia,fatty liver, weight-bearing arthralgia or dyspnoea,obstructive sleep apnoea syndrome) or obesity (BMI≥28 kg/m2) and with less than 5% body weight loss by diet and exercise 12 weeks or longer prior to screening were eligible for this study. \| 16 \| Placebo \| 9 mg, 10mg \| Mazdutide \| NCT04440345 \| 2022 \| Phase1b \| Ji,2022 \| \| adults (aged 18–75 years), overweight (BMI ≥ 24 kg/m2) accompanied by hyperphagia and/or at least one weight-related comorbidity (pre-dia- betes, hypertension, dyslipidaemia or fatty liver within 6 months before screening; weight-bearing joint pain; obesity-related dyspnoea or obstructive sleep apnoea syndrome), or with obesity (BMI ≥ 28 kg/m2); had body weight change less than 5% during the 2-week lead-in period \| 24 \| Placebo \| 3mg,4.5mg,6mg \| Mazdutide \| NCT04904913 \| 2023 \| Phase2 \| Ji,2023 \| \| aged at least 18 years, had a BMI 25 kg/m2, were diagnosed with type 2 diabetes and inad- equate blood glucose control (glycated hemoglobin A1c [HbA1c] level 7.0–10.5% [53–91 mmol/mol], inclusive), and had AST and ALT levels <3 times the upper limit of normal." \| 54 \| Placebo \| 100μg, 200μg, 300μg \| Cotadutide \| NCT03235050 \| 2021 \| Phase2b \| Nahra,2021 \| \| 18 to 75 years of age, have been diagnosed with T2D based on the(WHO), have an HbA1c value at screening of 7.0% and 10.5% and treated with diet and exercise alone or with a stable dose of metformin (either immediate release or extended release, 1000 mg/day and not more than the locally approved dose) for at least 3 months prior to screening/Visit 1. Have had a stable body weight for the 3 months prior to randomization (5 kg body weight gain and/or loss) Have a (BMI) of 25-50 kg/m2 at Visit 1 \| 36 \| Placebo \| 0.5 mg, 4 mg escalation, 4 mg, 8 mg slow escalation, 8 mg fast escalation,12 mg escalation \| Retatrutide \| NCT04867785 \| 2023 \| Phase2 \| Rosenstock,2023 \| \| adults (aged ≥18 to <75 years) with a BMI of 27 kg/m2 or greater, a stable bodyweight of 70 kg or greater (females) or 80 kg or greater (males), and with HbA1c less than 6·5% (without diabetes) at screening. Patients must have undergone at least one previous unsuccessful nonsurgical weight-loss attempt. \| 46 \| Placebo \| 0.6 mg, 2.4 mg, 3.6 mg, 4.8 mg \| Survodutide \| NCT04667377 \| 2024 \| Phase2 \| Roux,2024 \| \| patients of 10% or greater liver fat content by magnetic resonance imaging proton density fat fraction (MRI–PDFF) for participation in the MASLD substudy. \| 24 \| Placebo \| 1 mg, 4 mg, 8 mg,12 mg \| Retatrutide \| NCT04881760 \| 2024 \| Phase2 \| Sanyal,2024 \| \| Adults aged 20–70 years with type 2 diabetes for at least 3 months before screening, HbA1c level of 7·0–10·5% at lead-in and screening, BMI of 23–50 kg/m2, stable body weight (<5% change during the previous 3 months), and without advanced known possible complications of diabetes were eligible. Participants could not have used any glucose-lowering medication other than metforminwithin 3 months before screening. If taking a stable dose of metformin at study entry, participants must have been willing to maintain this dose during the trial. \| 12 \| Placebo \| 0.5 mg, 1.5 mg, 3 mg, 3/6 mg, 3/6/9/12 mg \| Retatrutide \| NCT04143802 \| 2022 \| Phase1b \| Urva,2022 \| \| adults aged 18–75 years who had been diagnosed at least 6 months earlier with type 2 diabetes that was inadequately controlled with diet and exercise alone or with stable metformin therapy 3 months before screening, with HbA1c of 7.0% to 10.5%  , BMI of 20 and <40 kg/m2 at screening and with stable body weight (change no more than 5%) during the previous 12 weeks before screening, a \| 20 \| Placebo \| 3 mg, 4.5 mg, 6 mg \| Mazdutide \| NCT03928379 \| 2024 \| Phase2 \| Zhang,2024 \|  Supplement 3: baseline characteristics of included studies  \| **Studlab** \| **Year** \| **n1** \| **Treat1,Dose** \| **Age (Mean ± SD)** \| **Male %** \| **BMI (Mean ± SD)** \| **weight (Mean ± SD)** \| **Waist circumference, cm (Mean ± SD)** \| **Hgb A1C % (Mean ± SD)** \| **n2** \| **Treat2,Dose** \| \| --- \| --- \| --- \| --- \| --- \| --- \| --- \| --- \| --- \| --- \| --- \| --- \| \| Jastreboff,2023 \| 2023 \| 268 \| Retatrutide 1 mg, 4 mg (ID 2 mg or ID 4 mg), 8 mg (ID 2 mg or ID 4 mg), and 12 mg (ID 2 mg) \| 48.2+12.7 \| 52 \| 37.3± 5.7 \| 107.7± 21.4 \| 115.5±14.7 \| 5.5±0.4 \| 70 \| Placebo \| \| Blüher,2023 \| 2023 \| 302 \| Survodutide 0.3 mg, 0.9 mg, 1.8 mg, and 2.7 mg qw, and 1.2 mg, 1.8 mg biw \| 57.3±9.8 \| 56.7 \| 33.9±6.0 \| 96.6±21.6 \| 110.3±18.2 \| 8.07±0.84 \| 109 \| Placebo \| \| Jungnik,2022 \| 2022 \| 36 \| Survodutide 16.39 mg, 38.24 mg, 42.60 mg \| 45.1±13.7 \| 68.9 \| 30.9±3.5 \| 94.1±15.0 \| NR \| NR \| 9 \| Placebo \| \| Yazawa,2023 \| 2023 \| 27 \| Survodutide 1.8 mg, 4.8 mg, 2.4 mg \| 34.2±7.6 \| 100 \| 25.2±1.8 \| 75.1±6.9 \| NR \| NR \| 9 \| Placebo \| \| Jiang,2022 \| 2022 \| 30 \| Mazdutide 3 mg, 4.5 mg, 6 mg \| 52.3±8.6 \| 62 \| 25.8±2.6 \| 69.5±11.1 \| NR \| 8.5±1.0 \| 12 \| Placebo \| \| Ji,2021 \| 2021 \| 24 \| Mazdutide 3 mg, 4.5 mg, 6 mg \| 34.1±16.0 \| 47.2 \| 30.6±3.09 \| 86.27±10.8 \| 98.9±10.60 \| 5.24±0.35 \| 12 \| Placebo \| \| Ji,2022 \| 2022 \| 16 \| Mazdutide 9 mg,10 mg \| 37±9.4 \| 29.2 \| 30.5±4.1 \| 81±13.8 \| 99.4±11.6 \| NR \| 8 \| Placebo \| \| Ji,2023 \| 2023 \| 186 \| Mazdutide 3 mg, 4.5 mg, 6 mg \| 35.5±9.4 \| 47.9 \| 31.8±4.2 \| 89.2±15 \| 104.2±10.4 \| 5.38±0.31 \| 62 \| Placebo \| \| Nahra,2021 \| 2021 \| 612 \| Cotadutide 100 μg, 200 μg, 300 μg \| 56.7±9.9 \| 46.28 \| 35.1±5.5 \| 99.8±20.4 \| NR \| 8.1±1.0 \| 222 \| Placebo \| \| Rosenstock,2023 \| 2023 \| 190 \| Retatrutide 0·5 mg, 4 mg escalation, 4 mg, 8 mg slow escalation, 8 mg fast escalation,12 mg escalation \| 56.2±9.7 \| 44 \| 35±6.3 \| 98.2±21.1 \| 111.8±15.9 \| 8.3±1.1 \| 91 \| Placebo \| \| Roux,2024 \| 2024 \| 307 \| Survodutide 0.6 mg, 2.4 mg, 3.6 mg, 4.8 mg \| 49.1±12.9 \| 32 \| 37·1±6·1 \| 105.7±20.4 \| 113·4±14·5 \| NR \| 77 \| Placebo \| \| Sanyal,2024 \| 2024 \| 79 \| Retatrutide1 mg, 4 mg, 8 mg,12 mg \| 46.6±12 \| 53.4 \| 38.4±5.2 \| 110.2±18.6 \| 118.3±13.4 \| 5.6±0.4 \| 19 \| Placebo \| \| Urva,2022 \| 2022 \| 52 \| Retatrutide 0.5 mg, 1.5 mg, 3 mg, 3/6 mg, 3/6/9/12 mg \| 58.4±7.4 \| 49 \| 32.1±5.1 \| 85.7±17.1 \| 106±12.2 \| 8.7±0.9 \| 20 \| Placebo \| \| Zhang,2024 \| 2024 \| 149 \| Mazdutide 3 mg, 4.5 mg, 6 mg \| 53.46±11.58 \| 59.2 \| 27.3±3.8 \| 75.5±14.6 \| 95.1±9.6 \| 8.1±0.9 \| 101 \| Placebo \| |
| --- | --- | --- | --- | --- | --- | --- | --- | --- | --- | --- | --- | --- | --- | --- | --- | --- | --- | --- | --- | --- | --- | --- | --- | --- | --- | --- | --- | --- | --- | --- | --- | --- | --- | --- | --- | --- | --- | --- | --- | --- | --- | --- | --- | --- | --- | --- | --- | --- | --- | --- | --- | --- | --- | --- | --- | --- | --- | --- | --- | --- | --- | --- | --- | --- | --- | --- | --- | --- | --- | --- | --- | --- | --- | --- | --- | --- | --- | --- | --- | --- | --- | --- | --- | --- | --- | --- | --- | --- | --- | --- | --- | --- | --- | --- | --- | --- | --- | --- | --- | --- | --- | --- | --- | --- | --- | --- | --- | --- | --- | --- | --- | --- | --- | --- | --- | --- | --- | --- | --- | --- | --- | --- | --- | --- | --- | --- | --- | --- | --- | --- | --- | --- | --- | --- | --- | --- | --- | --- | --- | --- | --- | --- | --- | --- | --- | --- | --- | --- | --- | --- | --- | --- | --- | --- | --- | --- | --- | --- | --- | --- | --- | --- | --- | --- | --- | --- | --- | --- | --- | --- | --- | --- | --- | --- | --- | --- | --- | --- | --- | --- | --- | --- | --- | --- | --- | --- | --- | --- | --- | --- | --- | --- | --- | --- | --- | --- | --- | --- | --- | --- | --- | --- | --- | --- | --- | --- | --- | --- | --- | --- | --- | --- | --- | --- | --- | --- | --- | --- | --- | --- | --- | --- | --- | --- | --- | --- | --- | --- | --- | --- | --- | --- | --- | --- | --- | --- | --- | --- | --- | --- | --- | --- | --- | --- | --- | --- | --- | --- | --- | --- | --- | --- | --- | --- | --- | --- | --- | --- | --- | --- | --- | --- | --- | --- | --- | --- | --- | --- | --- | --- | --- | --- | --- | --- | --- | --- | --- | --- | --- | --- | --- | --- | --- | --- | --- | --- | --- | --- | --- | --- | --- | --- | --- | --- | --- | --- | --- | --- | --- | --- | --- | --- | --- | --- | --- | --- | --- | --- | --- | --- | --- | --- | --- | --- | --- |

# Supplement 4: Publication bias -funnel plot- for each outcome

## S4.1: Publication bias -funnel plot- for absolute weight

**
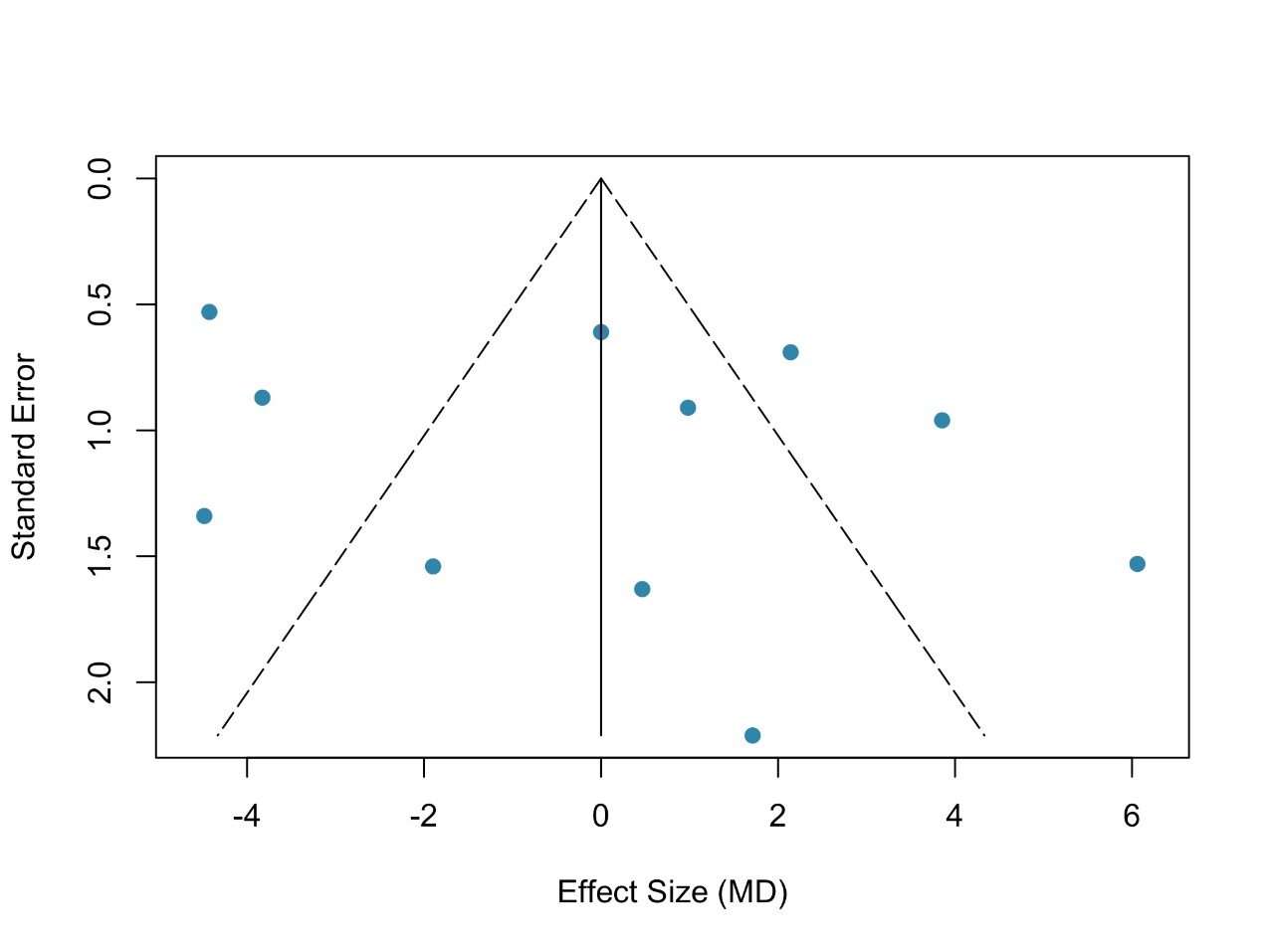
**

## S4.2: Publication bias -funnel plot- for percent weight

**
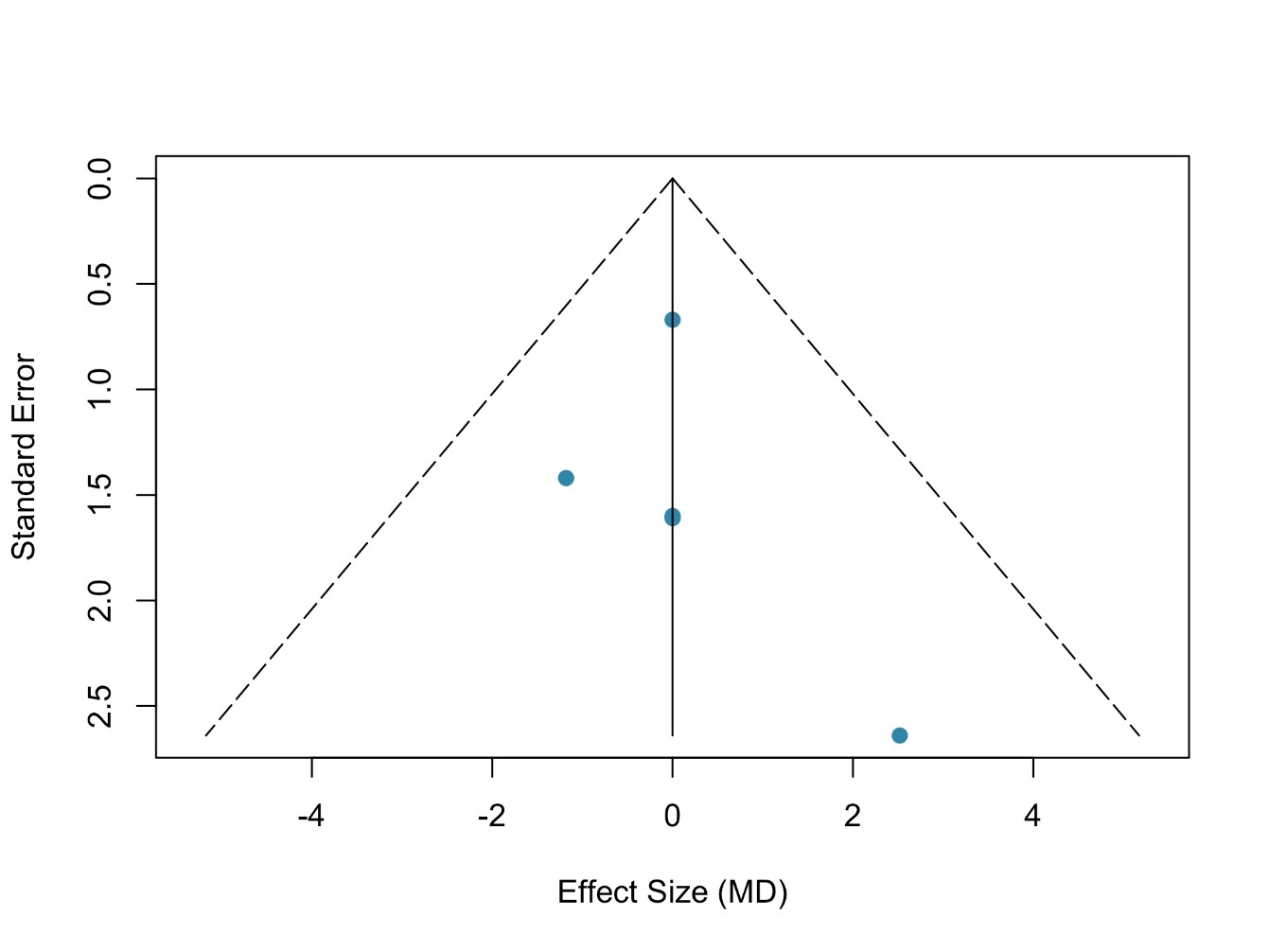
**

## S4.3: Publication bias -funnel plot- for HbA1c

*
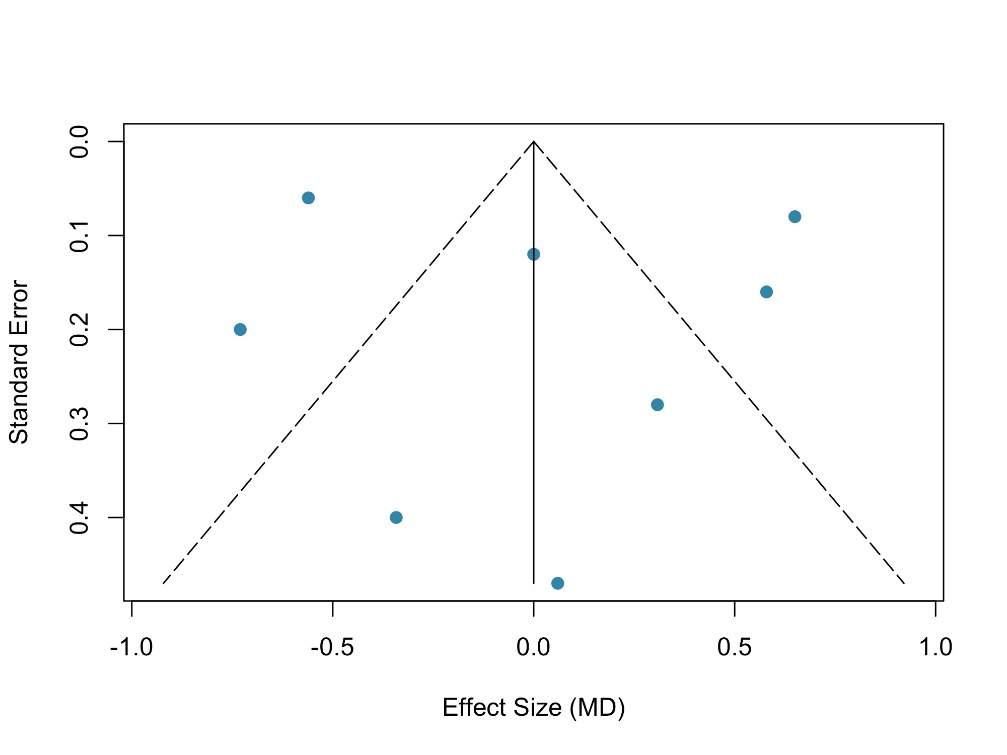
*

## S4.4: Publication bias -funnel plot- for adverse events

*
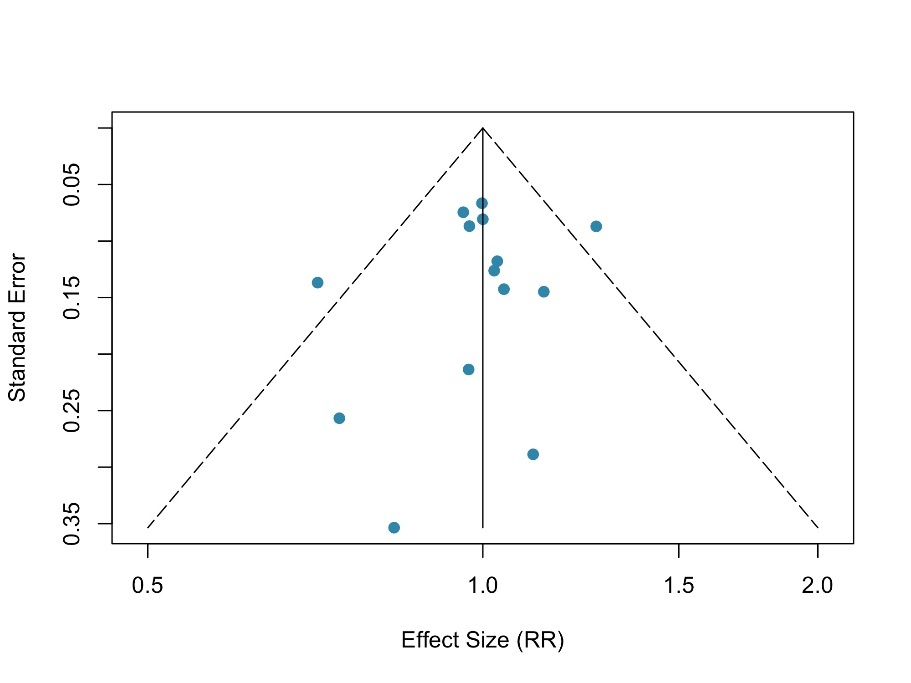
*

## S4.5: Publication bias -funnel plot- for discontinuation of treatment due to adverse events:

*
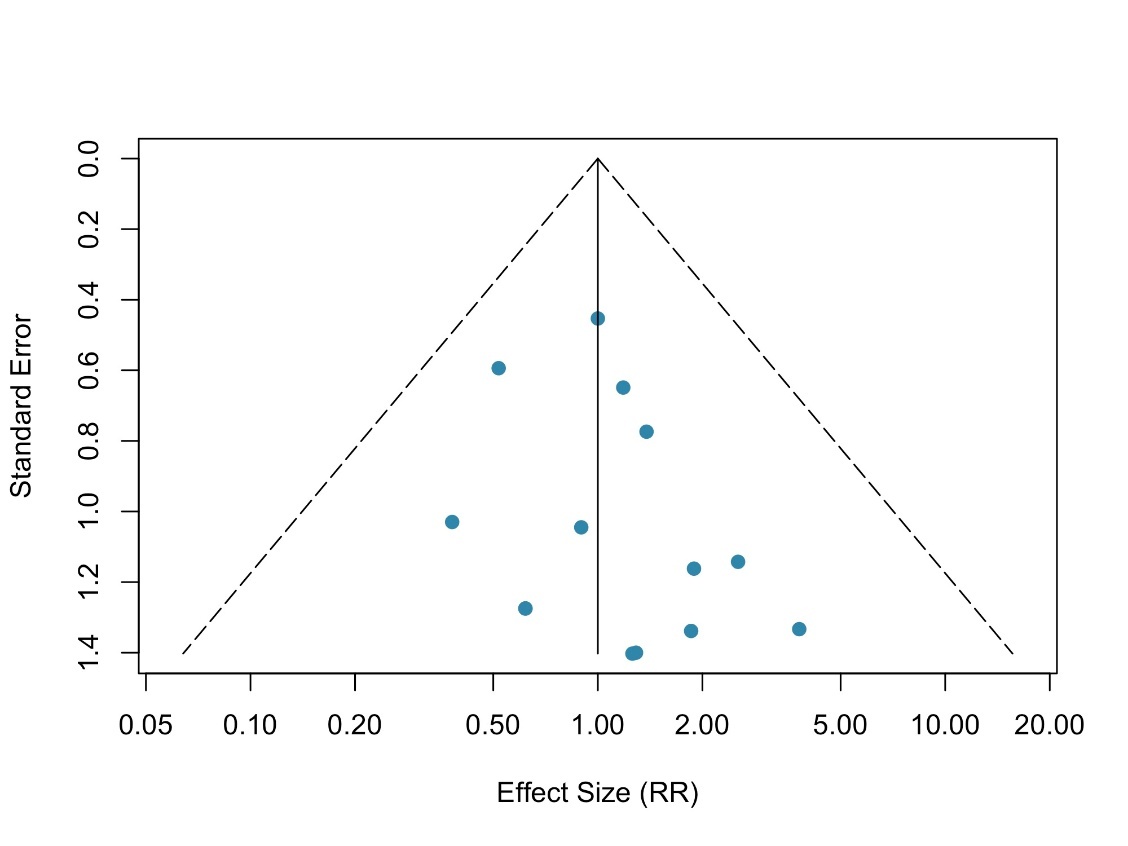
*

## S4.6: Publication bias -funnel plot- for vomiting:


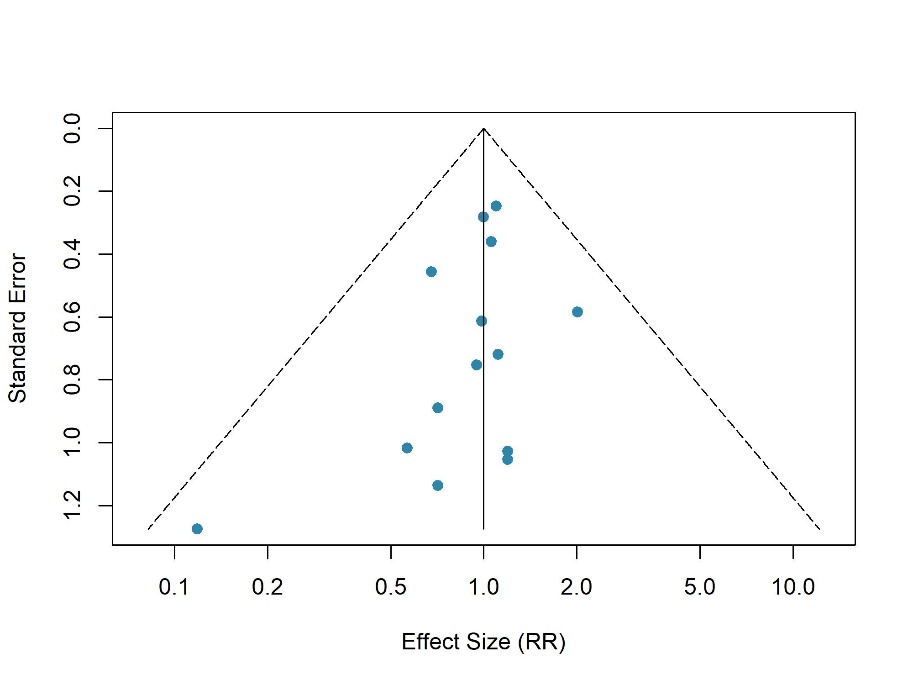


## S4.7: Publication bias -funnel plot- for Diarrhea:

## *
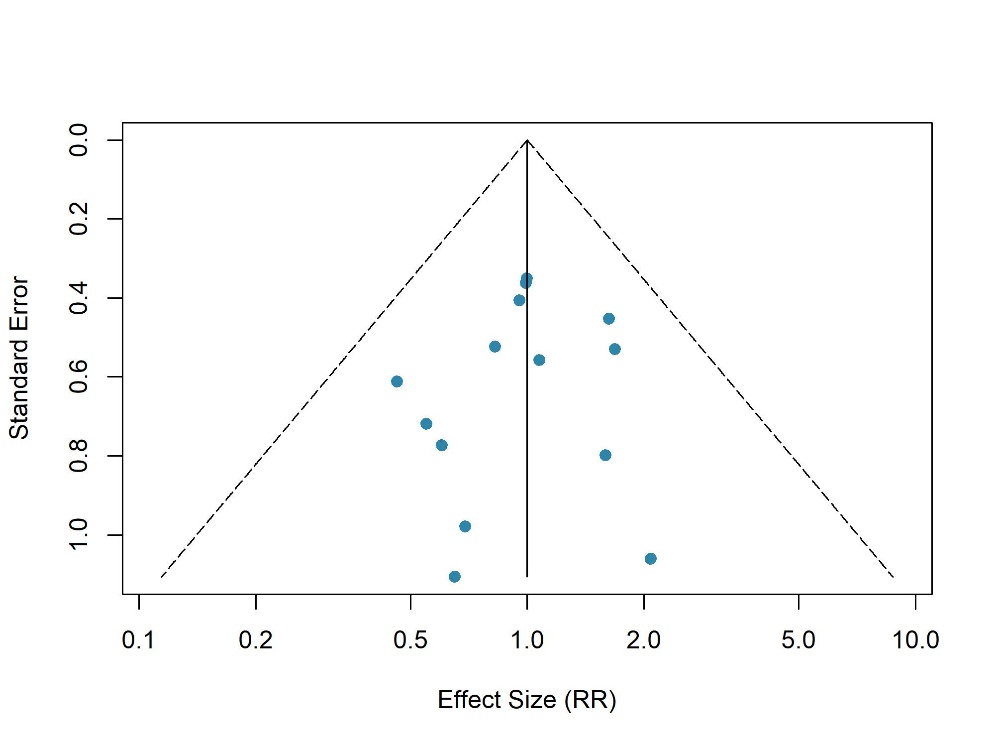
*

## S4.8: Publication bias -funnel plot- for Nausea:


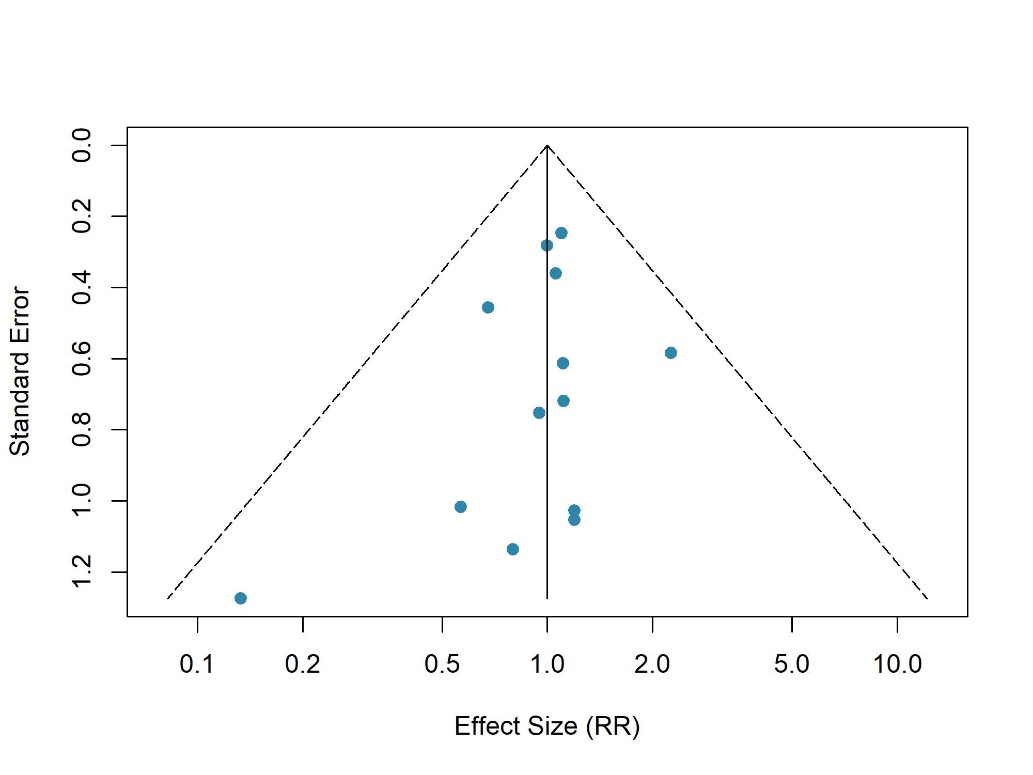


## S4.9: Publication bias -funnel plot- for Dyspepsia: *
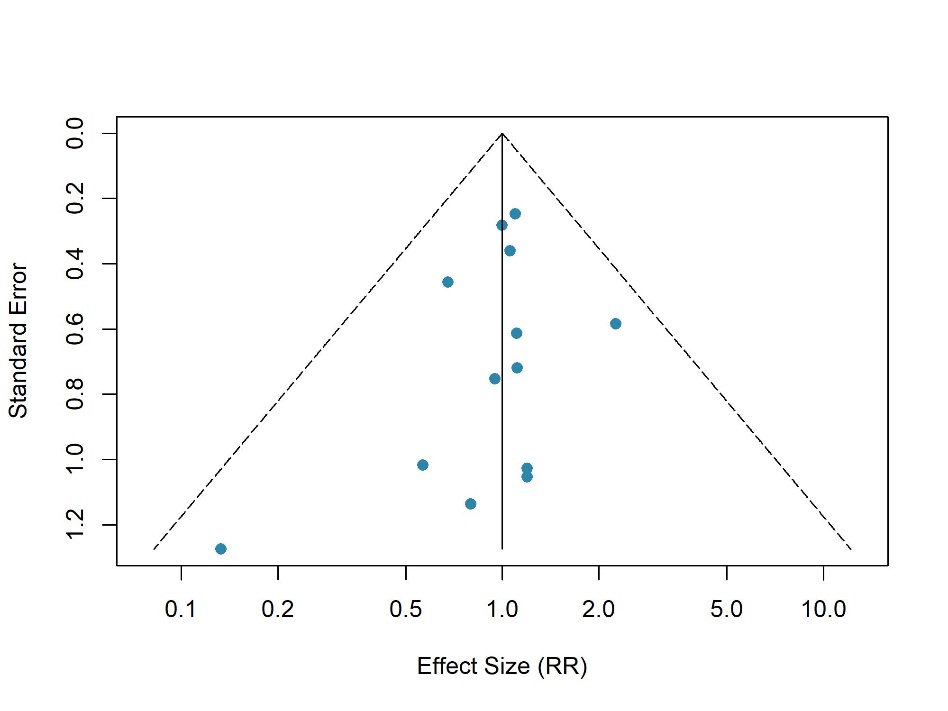
*

# Supplement 5: Certainty of evidence for each outcome

## S5.1: Certainty of evidence for absolute weight

| **Comparison** | **Within_Study_Bias** | **Reporting_Bias** | **Indirectness** | **Imprecision** | **Heterogeneity** | **Incoherence** | **Overall_Confidence** | **Reason_for_downgrade** |
| --- | --- | --- | --- | --- | --- | --- | --- | --- |
| Placebo:Survodutide | No concerns | Some concerns | No concerns | Some concerns | Major concerns | No concerns | Low | Reporting bias, Imprecision, Heterogeneity |
| Placebo:Retatrutide | Some concerns | Some concerns | No concerns | Some concerns | Major concerns | No concerns | Low | Within-study bias, Reporting bias, Imprecision, Heterogeneity |
| Mazdutide:Placebo | Some concerns | Some concerns | No concerns | Some concerns | Major concerns | No concerns | Low | Within-study bias, Reporting bias, Imprecision, Heterogeneity |
| Cotadutide:Placebo | Some concerns | Some concerns | No concerns | Major concerns | Major concerns | No concerns | Very low | Within-study bias, Reporting bias, Imprecision, Heterogeneity |
| Retatrutide:Survodutide | Some concerns | Some concerns | No concerns | Major concerns | Major concerns | No concerns | Very low | Within-study bias, Reporting bias, Imprecision, Heterogeneity |
| Mazdutide:Survodutide | Some concerns | Some concerns | No concerns | Major concerns | Major concerns | No concerns | Very low | Within-study bias, Reporting bias, Imprecision, Heterogeneity |
| Cotadutide:Survodutide | No concerns | Some concerns | No concerns | Major concerns | Major concerns | No concerns | Very low | Reporting bias, Imprecision, Heterogeneity |
| Mazdutide:Retatrutide | Some concerns | Some concerns | No concerns | Some concerns | Major concerns | No concerns | Low | Within-study bias, Reporting bias, Imprecision, Heterogeneity |
| Cotadutide:Retatrutide | Some concerns | Some concerns | No concerns | Some concerns | Major concerns | No concerns | Low | Within-study bias, Reporting bias, Imprecision, Heterogeneity |
| Cotadutide:Mazdutide | Some concerns | Some concerns | No concerns | Major concerns | Major concerns | No concerns | Very low | Within-study bias, Reporting bias, Imprecision, Heterogeneity |

## S5.2: Certainty of evidence for percent weight

| Comparison | Within_Study_Bias | Reporting_Bias | Indirectness | Imprecision | Heterogeneity | Incoherence | Overall_Confidence | Reason_for_downgrade |
| --- | --- | --- | --- | --- | --- | --- | --- | --- |
| Placebo:Retatrutide | No concerns | Some concerns | No concerns | Some concerns | Major concerns | No concerns | Low | Reporting bias, Imprecision, Heterogeneity |
| Mazdutide:Placebo | Some concerns | Some concerns | No concerns | Some concerns | Major concerns | No concerns | Low | Within-study bias, Reporting bias, Imprecision, Heterogeneity |
| Placebo:Survodutide | Major concerns | Some concerns | No concerns | Some concerns | Major concerns | No concerns | Very low | Within-study bias, Reporting bias, Imprecision, Heterogeneity |
| Cotadutide:Placebo | Some concerns | Some concerns | No concerns | Some concerns | Major concerns | No concerns | Low | Within-study bias, Reporting bias, Imprecision, Heterogeneity |
| Mazdutide:Retatrutide | Some concerns | Some concerns | No concerns | Some concerns | Major concerns | No concerns | Low | Within-study bias, Reporting bias, Imprecision, Heterogeneity |
| Retatrutide:Survodutide | Some concerns | Some concerns | No concerns | Some concerns | Major concerns | No concerns | Low | Within-study bias, Reporting bias, Imprecision, Heterogeneity |
| Cotadutide:Retatrutide | Some concerns | Some concerns | No concerns | Some concerns | Major concerns | No concerns | Low | Within-study bias, Reporting bias, Imprecision, Heterogeneity |
| Mazdutide:Survodutide | Some concerns | Some concerns | No concerns | Some concerns | Major concerns | No concerns | Low | Within-study bias, Reporting bias, Imprecision, Heterogeneity |
| Cotadutide:Mazdutide | Some concerns | Some concerns | No concerns | Major concerns | Major concerns | No concerns | Very low | Within-study bias, Reporting bias, Imprecision, Heterogeneity |
| Cotadutide:Survodutide | Some concerns | Some concerns | No concerns | Some concerns | Major concerns | No concerns | Low | Within-study bias, Reporting bias, Imprecision, Heterogeneity |

## S5.3: Certainty of evidence for HbA1c

| **Comparison** | **Within_Study_Bias** | **Reporting_Bias** | **Indirectness** | **Imprecision** | **Heterogeneity** | **Incoherence** | **Overall_Confidence** | **Reason_for_downgrade** |
| --- | --- | --- | --- | --- | --- | --- | --- | --- |
| Placebo:Survodutide | No concerns | Some concerns | No concerns | Major concerns | Major concerns | No concerns | Very low | Reporting bias, Imprecision, Heterogeneity |
| Mazdutide:Placebo | Some concerns | Some concerns | No concerns | Major concerns | Major concerns | No concerns | Very low | Within-study bias, Reporting bias, Imprecision, Heterogeneity |
| Cotadutide:Placebo | Some concerns | Some concerns | No concerns | Major concerns | Major concerns | No concerns | Very low | Within-study bias, Reporting bias, Imprecision, Heterogeneity |
| Placebo:Retatrutide | Some concerns | Some concerns | No concerns | Some concerns | Major concerns | No concerns | Low | Within-study bias, Reporting bias, Imprecision, Heterogeneity |
| Mazdutide:Survodutide | Some concerns | Some concerns | No concerns | Major concerns | Major concerns | No concerns | Very low | Within-study bias, Reporting bias, Imprecision, Heterogeneity |
| Cotadutide:Survodutide | Some concerns | Some concerns | No concerns | Major concerns | Major concerns | No concerns | Very low | Within-study bias, Reporting bias, Imprecision, Heterogeneity |
| Retatrutide:Survodutide | Some concerns | Some concerns | No concerns | Major concerns | Major concerns | No concerns | Very low | Within-study bias, Reporting bias, Imprecision, Heterogeneity |
| Cotadutide:Mazdutide | Some concerns | Some concerns | No concerns | Major concerns | Major concerns | No concerns | Very low | Within-study bias, Reporting bias, Imprecision, Heterogeneity |
| Mazdutide:Retatrutide | Some concerns | Some concerns | No concerns | Major concerns | Major concerns | No concerns | Very low | Within-study bias, Reporting bias, Imprecision, Heterogeneity |
| Cotadutide:Retatrutide | Some concerns | Some concerns | No concerns | Major concerns | Major concerns | No concerns | Very low | Within-study bias, Reporting bias, Imprecision, Heterogeneity |

## S5.4: Certainty of evidence for adverse events

| Comparison | Within_Study_Bias | Reporting_Bias | Indirectness | Imprecision | Heterogeneity | Incoherence | Overall_Confidence | Reason_for_downgrade |
| --- | --- | --- | --- | --- | --- | --- | --- | --- |
| Placebo:Survodutide | No concerns | Some concerns | No concerns | Major concerns | Major concerns | No concerns | Very low | Reporting bias, Imprecision, Heterogeneity |
| Mazdutide:Placebo | Some concerns | Some concerns | No concerns | Major concerns | Major concerns | No concerns | Very low | Within-study bias, Reporting bias, Imprecision, Heterogeneity |
| Cotadutide:Placebo | Some concerns | Some concerns | No concerns | Major concerns | Major concerns | No concerns | Very low | Within-study bias, Reporting bias, Imprecision, Heterogeneity |
| Placebo:Retatrutide | Some concerns | Some concerns | No concerns | Some concerns | Major concerns | No concerns | Low | Within-study bias, Reporting bias, Imprecision, Heterogeneity |
| Mazdutide:Survodutide | Some concerns | Some concerns | No concerns | Major concerns | Major concerns | No concerns | Very low | Within-study bias, Reporting bias, Imprecision, Heterogeneity |
| Cotadutide:Survodutide | Some concerns | Some concerns | No concerns | Major concerns | Major concerns | No concerns | Very low | Within-study bias, Reporting bias, Imprecision, Heterogeneity |
| Retatrutide:Survodutide | Some concerns | Some concerns | No concerns | Major concerns | Major concerns | No concerns | Very low | Within-study bias, Reporting bias, Imprecision, Heterogeneity |
| Cotadutide:Mazdutide | Some concerns | Some concerns | No concerns | Major concerns | Major concerns | No concerns | Very low | Within-study bias, Reporting bias, Imprecision, Heterogeneity |
| Mazdutide:Retatrutide | Some concerns | Some concerns | No concerns | Major concerns | Major concerns | No concerns | Very low | Within-study bias, Reporting bias, Imprecision, Heterogeneity |
| Cotadutide:Retatrutide | Some concerns | Some concerns | No concerns | Major concerns | Major concerns | No concerns | Very low | Within-study bias, Reporting bias, Imprecision, Heterogeneity |

## S5.5: Certainty of evidence for treatment discontinuation due to adverse events

| Comparison | Within_Study_Bias | Reporting_Bias | Indirectness | Imprecision | Heterogeneity | Incoherence | Overall_Confidence | Reason_for_downgrade |
| --- | --- | --- | --- | --- | --- | --- | --- | --- |
| Placebo:Survodutide | Some concerns | Some concerns | No concerns | Major concerns | No concerns | No concerns | Low | Within-study bias, Reporting bias, Imprecision |
| Placebo:Retatrutide | Some concerns | Some concerns | No concerns | Major concerns | No concerns | No concerns | Low | Within-study bias, Reporting bias, Imprecision |
| Mazdutide:Placebo | Some concerns | Some concerns | No concerns | Major concerns | No concerns | No concerns | Low | Within-study bias, Reporting bias, Imprecision |
| Cotadutide:Placebo | Some concerns | Some concerns | No concerns | No concerns | No concerns | No concerns | Moderate | Within-study bias, Reporting bias |
| Retatrutide:Survodutide | Some concerns | Some concerns | No concerns | Major concerns | No concerns | No concerns | Low | Within-study bias, Reporting bias, Imprecision |
| Mazdutide:Survodutide | Some concerns | Some concerns | No concerns | Major concerns | No concerns | No concerns | Low | Within-study bias, Reporting bias, Imprecision |
| Cotadutide:Survodutide | Some concerns | Some concerns | No concerns | Some concerns | No concerns | No concerns | Low | Within-study bias, Reporting bias, Imprecision |
| Mazdutide:Retatrutide | Some concerns | Some concerns | No concerns | Major concerns | No concerns | No concerns | Low | Within-study bias, Reporting bias, Imprecision |
| Cotadutide:Retatrutide | Some concerns | Some concerns | No concerns | Major concerns | No concerns | No concerns | Low | Within-study bias, Reporting bias, Imprecision |
| Cotadutide:Mazdutide | Some concerns | Some concerns | No concerns | Some concerns | No concerns | No concerns | Low | Within-study bias, Reporting bias, Imprecision |

# Supplement 6. Network meta-analysis results (league)

## 6.1. Network meta-analysis results for change in absolute weight (kg)

|  | Cotadutide | Mazdutide | Placebo | Retatrutide | Survodutide |
| --- | --- | --- | --- | --- | --- |
| Cotadutide | Cotadutide | . | -3.41 [-11.63; 4.81] | . | . |
| Mazdutide | 3.06 [ -6.19; 12.31] | Mazdutide | -6.47 [-10.71; -2.24] | . | . |
| Placebo | -3.41 [-11.63; 4.81] | -6.47 [-10.71; -2.24] | Placebo | 13.45 [ 8.51; 18.38] | 10.74 [ 5.80; 15.67] |
| Retatrutide | 10.04 [ 0.44; 19.63] | 6.97 [ 0.47; 13.47] | 13.45 [ 8.51; 18.38] | Retatrutide | . |
| Survodutide | 7.33 [ -2.26; 16.92] | 4.27 [ -2.24; 10.77] | 10.74 [ 5.80; 15.67] | -2.71 [ -9.68; 4.27] | Survodutide |

## 6.2. Network meta-analysis results for change in weight percent

|  | Cotadutide | Mazdutide | Placebo | Retatrutide | Survodutide |
| --- | --- | --- | --- | --- | --- |
| Cotadutide | Cotadutide | . | -4.34 [ -7.62; -1.06] | . | . |
| Mazdutide | 0.06 [ -5.39; 5.51] | Mazdutide | -4.40 [ -8.76; -0.04] | . | . |
| Placebo | -4.34 [ -7.62; -1.06] | -4.40 [ -8.76; -0.04] | Placebo | 23.28 [ 19.90; 26.66] | 13.83 [ 9.49; 18.17] |
| Retatrutide | 18.94 [ 14.23; 23.65] | 18.88 [ 13.37; 24.40] | 23.28 [ 19.90; 26.66] | Retatrutide | . |
| Survodutide | 9.49 [ 4.05; 14.93] | 9.43 [ 3.28; 15.58] | 13.83 [ 9.49; 18.17] | -9.45 [-14.95; -3.95] | Survodutide |

## 6.3. Network meta-analysis results for Change from baseline in HbA1c

|  | Cotadutide | Mazdutide | Placebo | Retatrutide | Survodutide |
| --- | --- | --- | --- | --- | --- |
| Cotadutide | Cotadutide | . | -0.74 [-2.35; 0.87] | . | . |
| Mazdutide | 0.11 [-1.77; 1.99] | Mazdutide | -0.85 [-1.82; 0.12] | . | . |
| Placebo | -0.74 [-2.35; 0.87] | -0.85 [-1.82; 0.12] | Placebo | 1.54 [ 0.32; 2.76] | 0.95 [-0.19; 2.09] |
| Retatrutide | 0.80 [-1.22; 2.82] | 0.69 [-0.87; 2.25] | 1.54 [ 0.32; 2.76] | Retatrutide | . |
| Survodutide | 0.21 [-1.76; 2.18] | 0.10 [-1.39; 1.60] | 0.95 [-0.19; 2.09] | -0.59 [-2.26; 1.08] | Survodutide |

## 6.4. Network meta-analysis results for adverse events:

|  | Cotadutide | Mazdutide | Placebo | Retatrutide | Survodutide |
| --- | --- | --- | --- | --- | --- |
| Cotadutide | Cotadutide | . | 1.31 [1.01; 1.69] | . | . |
| Mazdutide | 1.09 [0.80; 1.47] | Mazdutide | 1.20 [1.02; 1.42] | . | . |
| Placebo | 1.31 [1.01; 1.69] | 1.20 [1.02; 1.42] | Placebo | 0.78 [0.66; 0.93] | 0.86 [0.75; 0.99] |
| Retatrutide | 1.02 [0.75; 1.39] | 0.94 [0.74; 1.19] | 0.78 [0.66; 0.93] | Retatrutide | . |
| Survodutide | 1.13 [0.84; 1.51] | 1.04 [0.83; 1.29] | 0.86 [0.75; 0.99] | 1.10 [0.88; 1.37] | Survodutide |

##

## 6.5. Network meta-analysis results for treatment discontinuation due to adverse events

|  | Cotadutide | Mazdutide | Placebo | Retatrutide | Survodutide |
| --- | --- | --- | --- | --- | --- |
| Cotadutide | Cotadutide | . | 4.81 [1.98; 11.70] | . | . |
| Mazdutide | 5.96 [1.37; 25.87] | Mazdutide | 0.81 [0.25; 2.60] | . | . |
| Placebo | 4.81 [1.98; 11.70] | 0.81 [0.25; 2.60] | Placebo | 0.21 [0.08; 0.55] | 0.26 [0.12; 0.57] |
| Retatrutide | 1.02 [0.28; 3.73] | 0.17 [0.04; 0.77] | 0.21 [0.08; 0.55] | Retatrutide | . |
| Survodutide | 1.27 [0.39; 4.09] | 0.21 [0.05; 0.86] | 0.26 [0.12; 0.57] | 1.24 [0.37; 4.21] | Survodutide |

# Supplement 7: Network meta-analysis results for each outcome

# S7.1: Network meta-analysis results for absolute weight loss


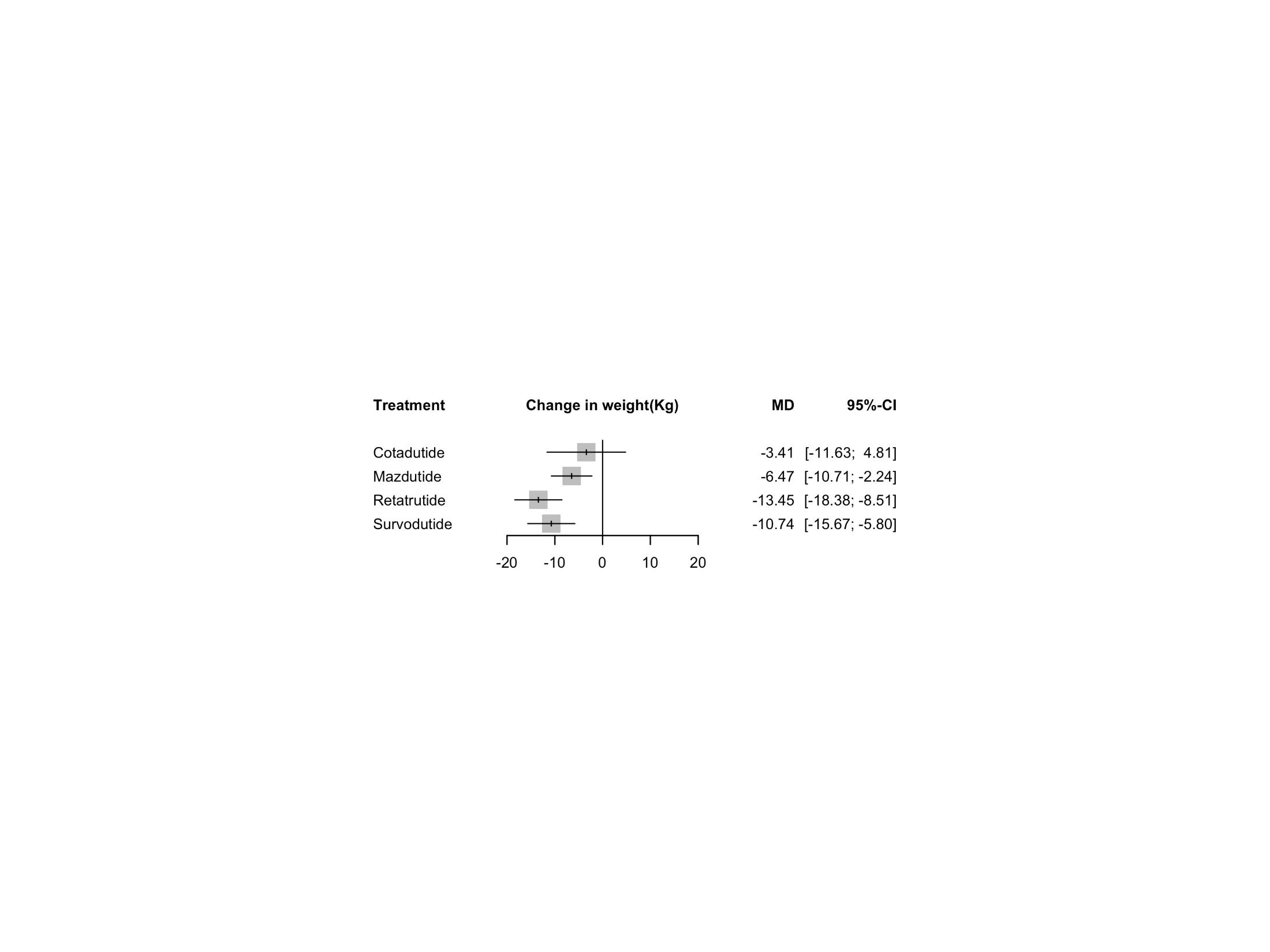


# S7.2: Network meta-analysis results for Percent weight changes

# *
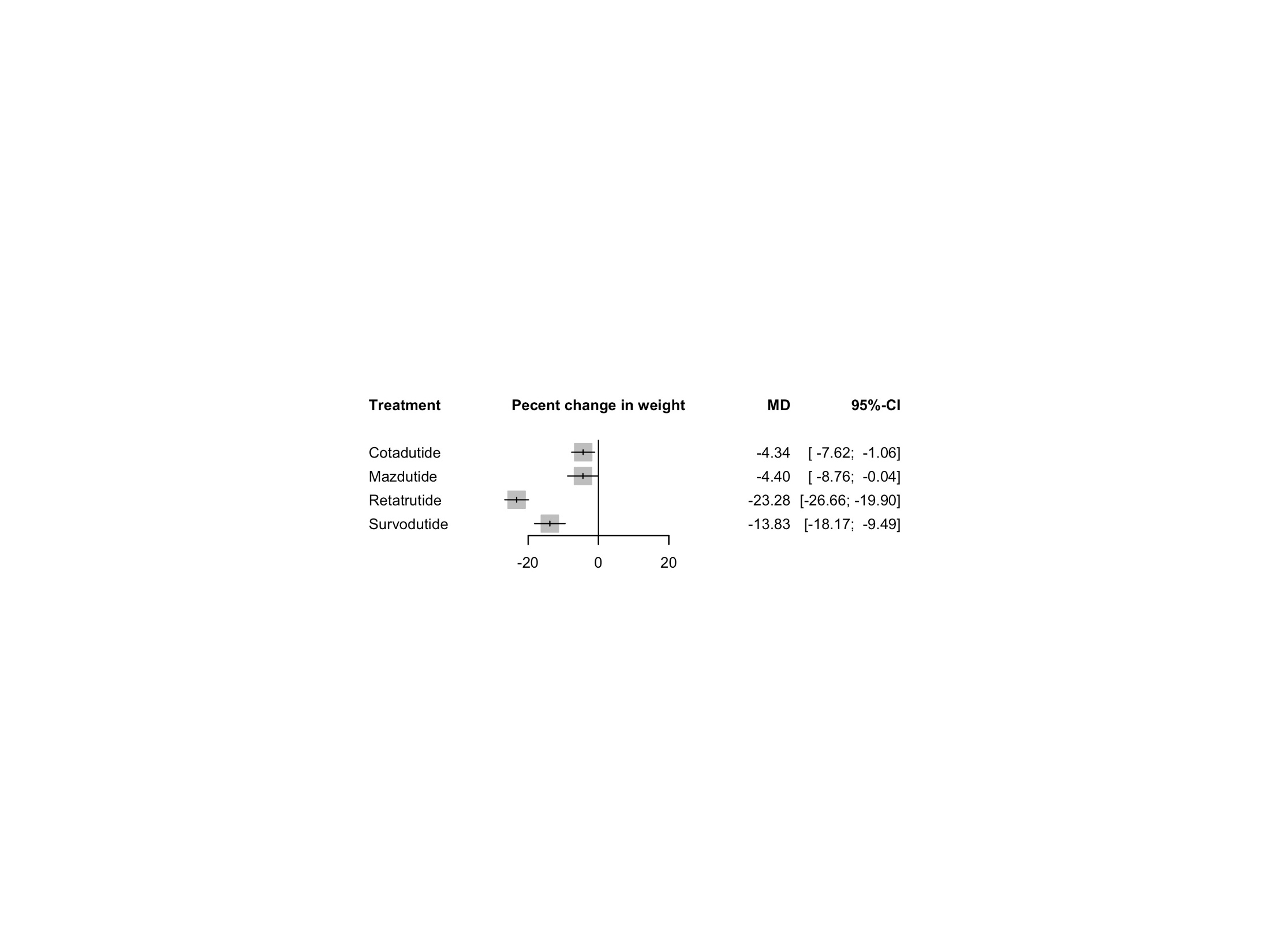
*

# S7.3: Network meta-analysis results for HbA1c

# *
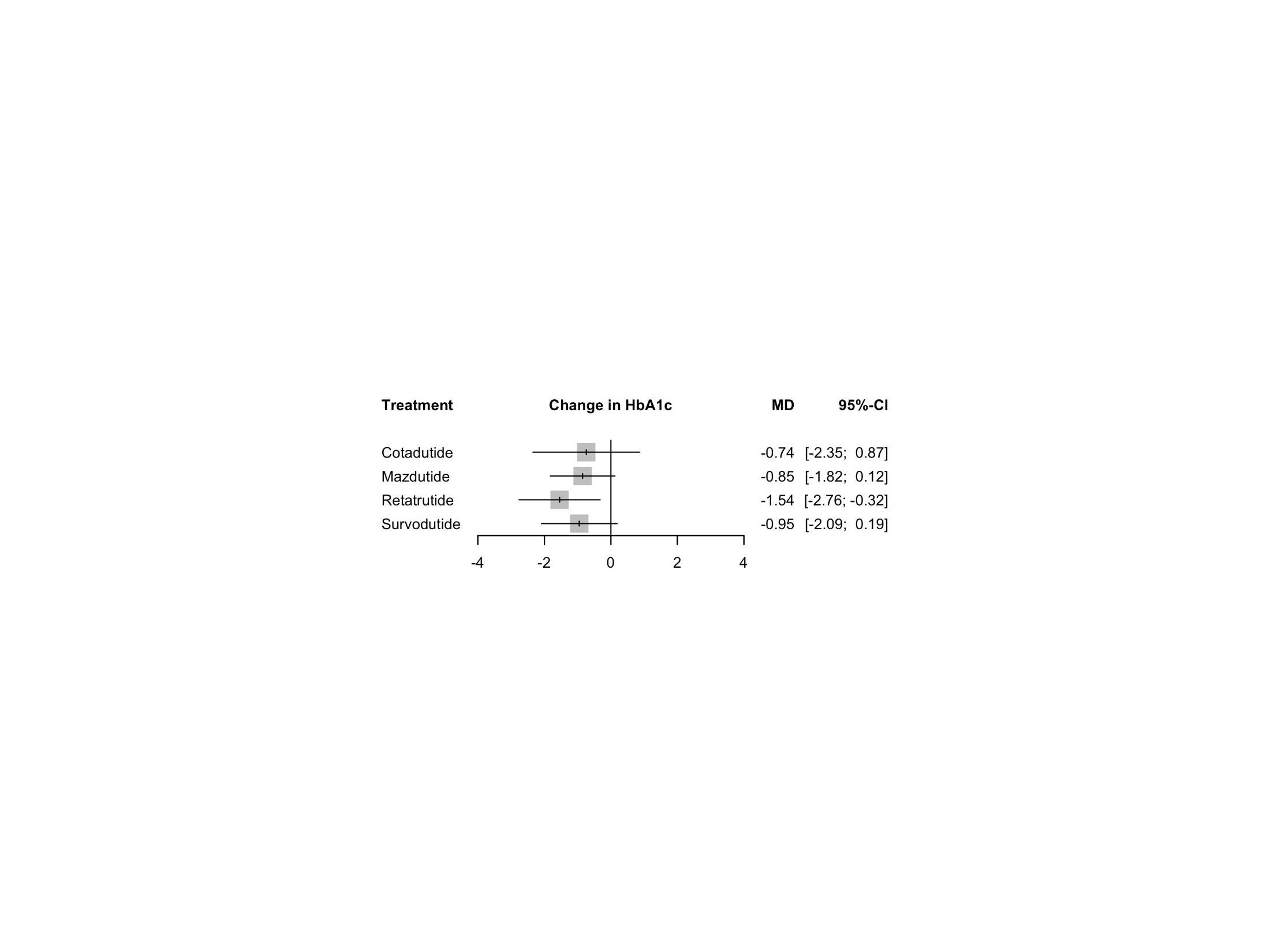
*

# S7.4: Network meta-analysis results for adverse events

# *
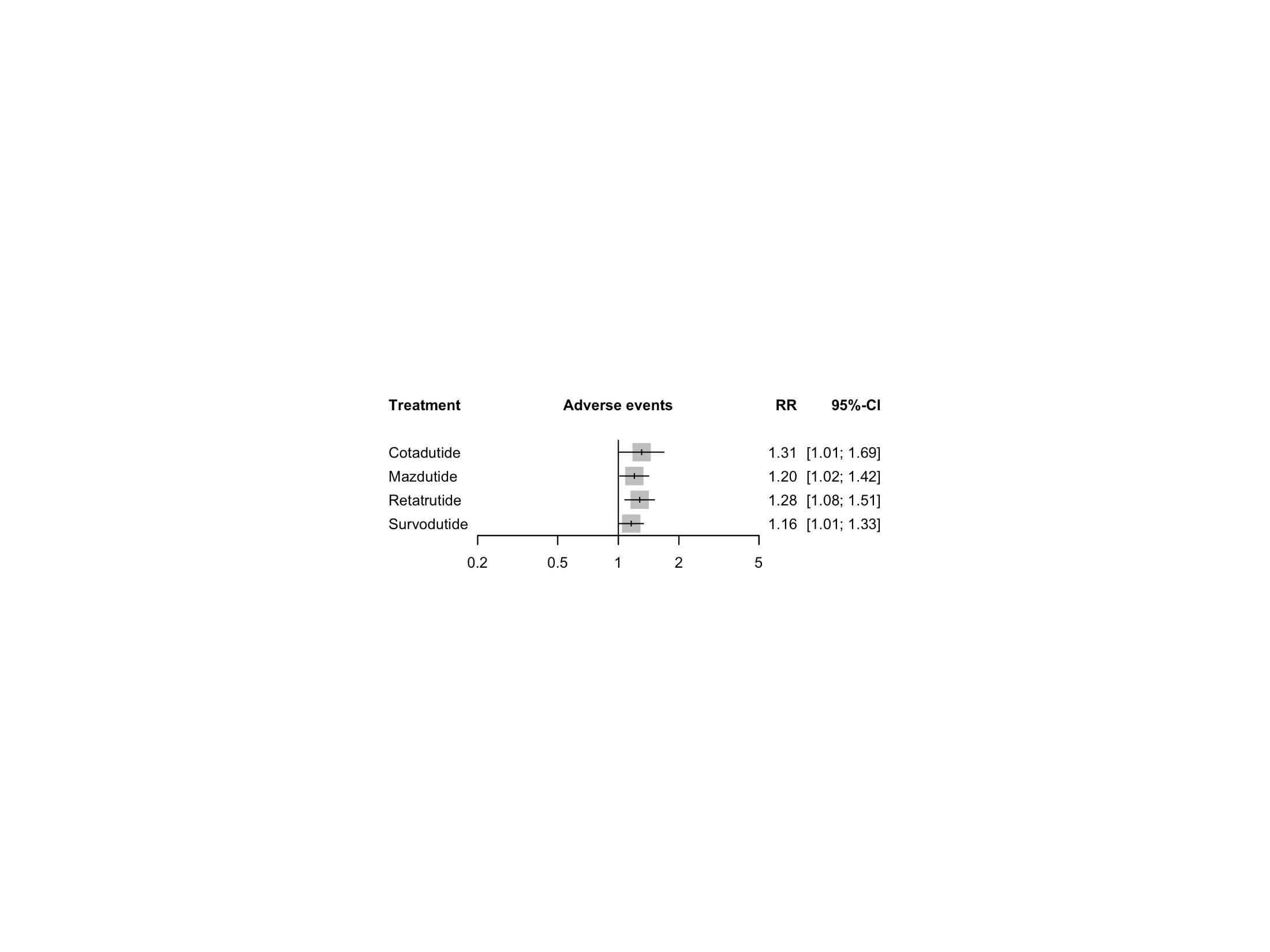
*

# S7.5: Network meta-analysis results for treatment discontinuation due to adverse events:


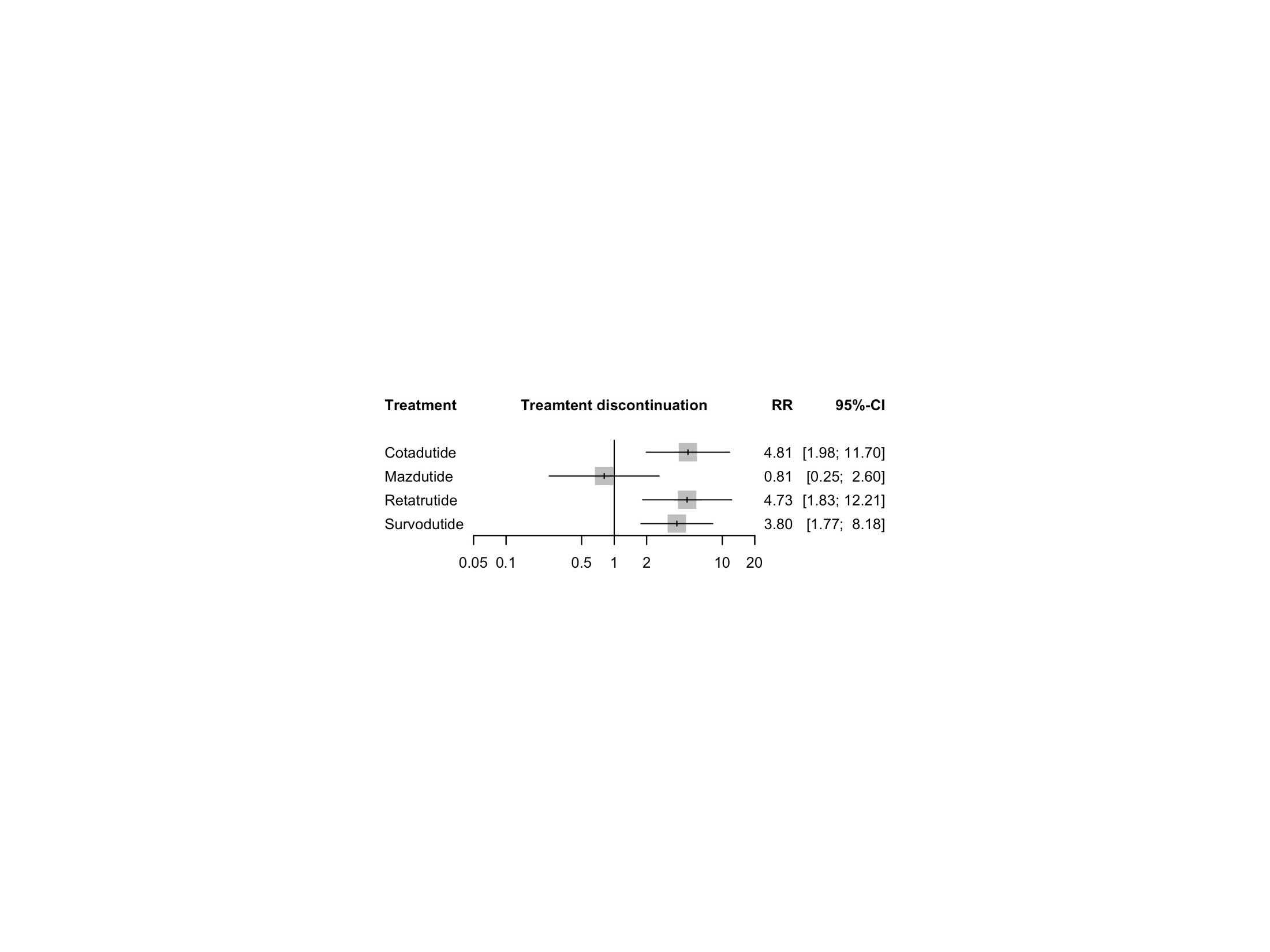


# Supplement 8: Network geometry for each outcome

## S8.1 Network geometry for absolute weight:


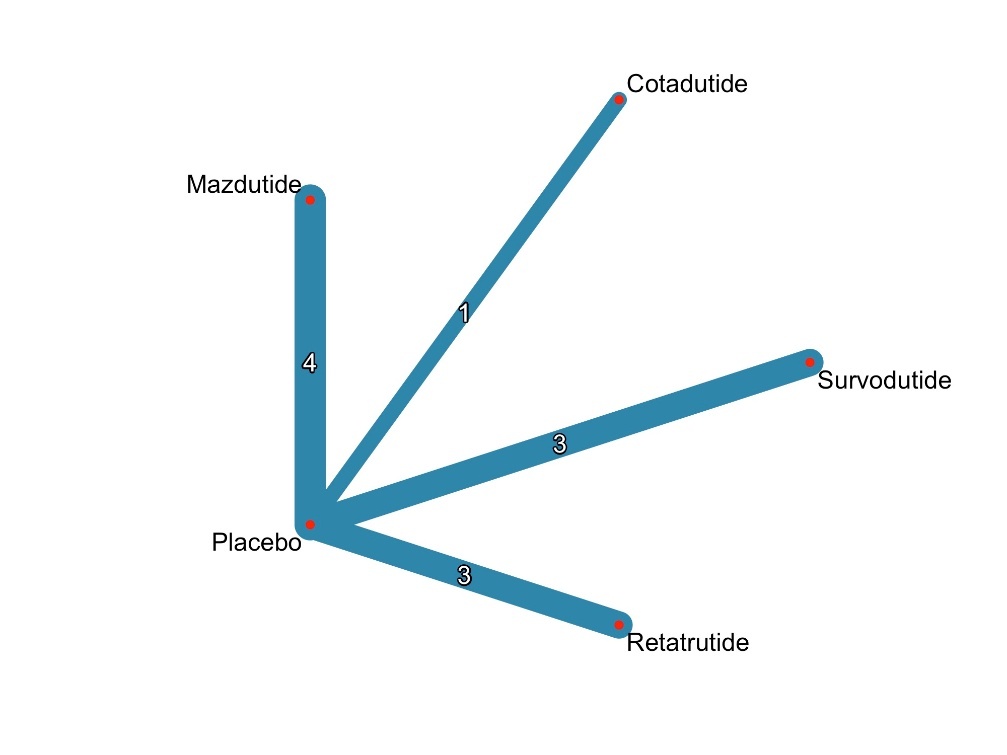


## S8.2 Network geometry for percent weight:

#
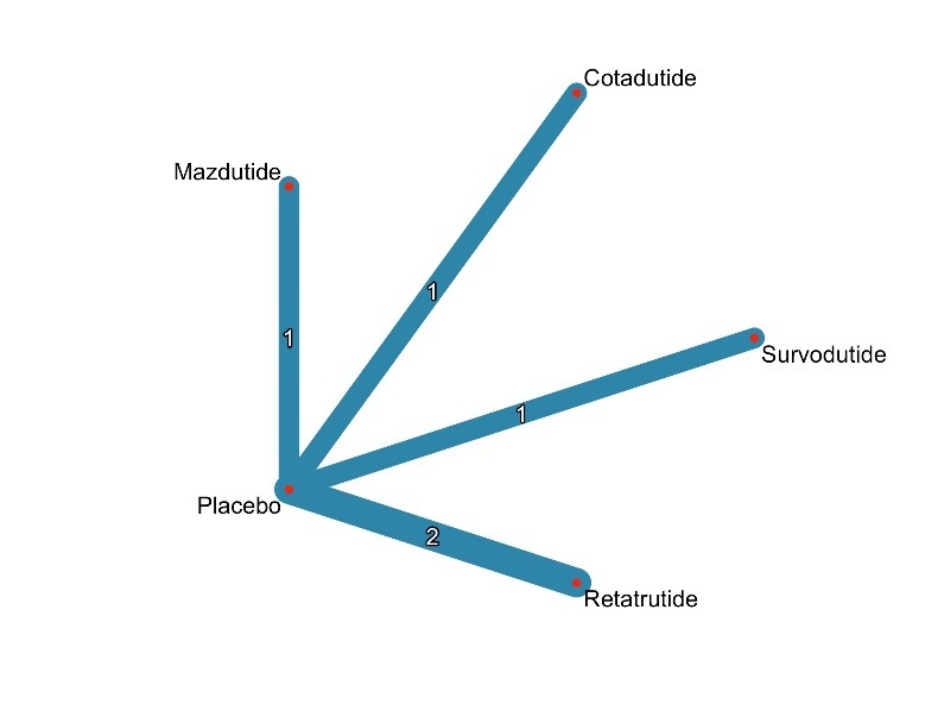


## S8.3 Network geometry for HbA1c


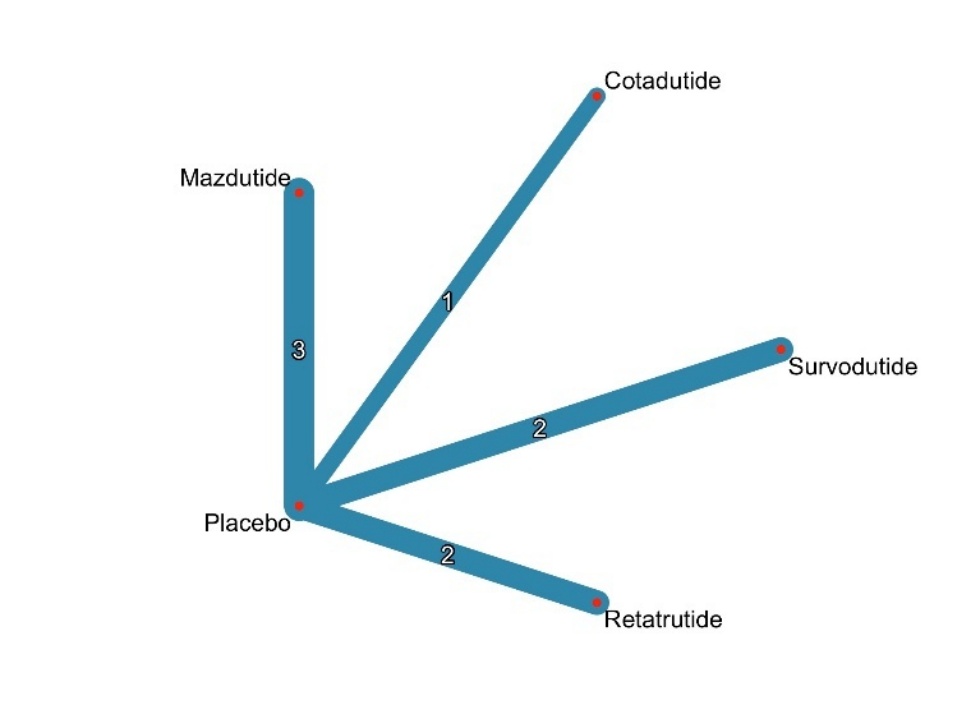


## S8.4 Network geometry for adverse events

*
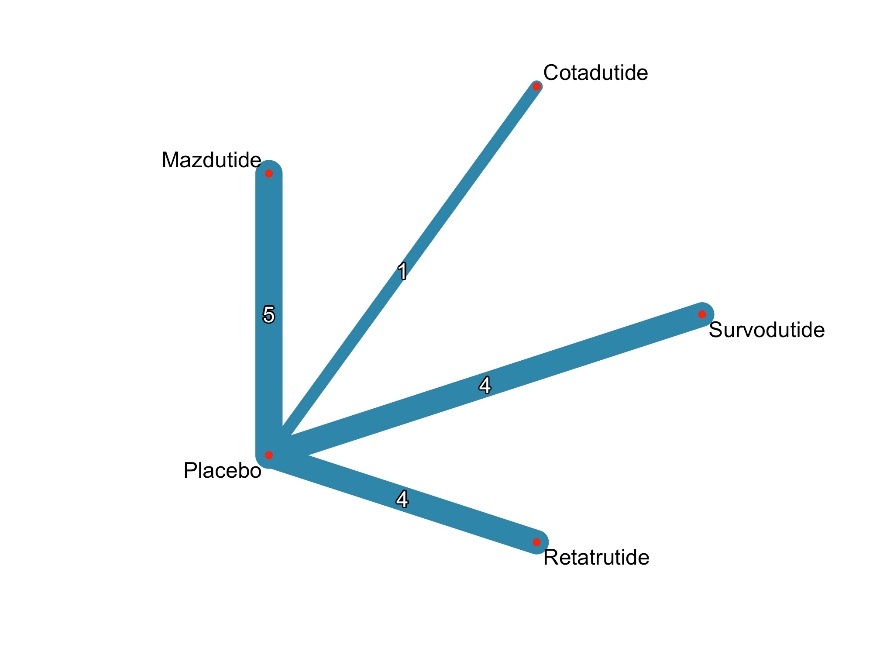
*

## S8.5 Network geometry for treatment discontinuation due to adverse events

*
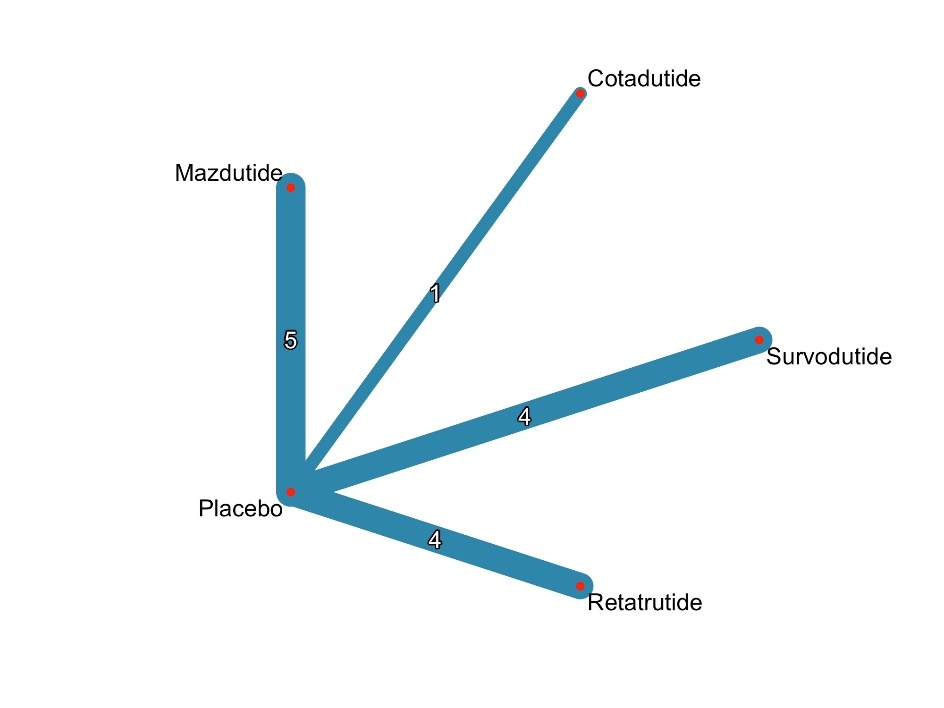
*

# Supplement 9. Risk of bias assessment of included trials for each outcome

S9.1 Change in absolute weight

| **Study ID** | **Randomization process** | **Deviations from intended intervention** | **Missing outcome data** | **Measurement of the outcome** | **Selection of the reported result** | **Overall bias** |
| --- | --- | --- | --- | --- | --- | --- |
| **Jastreboff,2023** | Low | Low | Some Concern | Low | Some Concern | Some Concern |
| **Blüher,2023** | Low | Low | Low | Low | Low | Low |
| **Yazawa,2023** | Low | Low | Low | Low | Low | Low |
| **Jiang,2022** | Low | Low | Low | Low | Low | Low |
| **Ji,2021** | Low | Low | Low | Low | Low | Some Concern |
| **Ji,2022** | Low | Low | Low | Low | Some Concern | Some Concern |
| **Ji,2023** | Low | Low | Low | Low | Some Concern | Some Concern |
| **Nahra,2021** | Low | Low | Some Concern | Low | Some Concern | Some Concern |
| **Rosenstock,2023** | Low | Low | Low | Low | Some Concern | Some Concern |
| **Urva,2022** | Low | Low | Low | Low | Some Concern | Some Concern |
| **Zhang,2024** | Low | Low | Low | Low | Some Concern | Low |

S9.2 Change in HbA1c

| **Study ID** | **Randomization process** | **Deviations from intended intervention** | **Missing outcome data** | **Measurement of the outcome** | **Selection of the reported result** | **Overall bias** |
| --- | --- | --- | --- | --- | --- | --- |
| **Jastreboff,2023** | Low | Low | Low | Low | Low | Low |
| **Blüher,2023** | Low | Low | Low | Low | Low | Low |
| **Yazawa,2023** | Low | Low | Low | Low | Low | Low |
| **Jiang,2022** | Low | Low | Low | Low | Low | Low |
| **Ji,2021** | Low | Low | Low | Low | Low | Some Concern |
| **Ji,2022** | Low | Low | Low | Low | Some Concern | Some Concern |
| **Ji,2023** | Low | Low | Low | Low | Some Concern | Some Concern |
| **Nahra,2021** | Low | Low | Some Concern | Low | Some Concern | Some Concern |
| **Rosenstock,2023** | Low | Low | Low | Low | Some Concern | Some Concern |
| **Urva,2022** | Low | Low | Low | Low | Some Concern | Some Concern |
| **Zhang,2024** | Low | Low | Low | Low | Some Concern | Low |
|  |  |  |  |  |  |  |

S9.3 Adverse Events

| **Study ID** | **Randomization process** | **Deviations from intended intervention** | **Missing outcome data** | **Measurement of the outcome** | **Selection of the reported result** | **Overall bias** |
| --- | --- | --- | --- | --- | --- | --- |
| **Jastreboff,2023** | Low | Low | Low | Some Concern | Low | Low |
| **Blüher,2023** | Low | Low | Low | Low | Low | Low |
| **Yazawa,2023** | Low | Low | Low | Low | Low | Low |
| **Jiang,2022** | Low | Low | Low | Low | Low | Low |
| **Ji,2021** | Low | Low | Low | Low | Low | Some Concern |
| **Ji,2022** | Low | Low | Low | Low | Some Concern | Some Concern |
| **Ji,2023** | Low | Low | Low | Low | Some Concern | Some Concern |
| **Nahra,2021** | Low | Low | Some Concern | Low | Some Concern | Some Concern |
| **Rosenstock,2023** | Low | Low | Low | Low | Some Concern | Some Concern |
| **Urva,2022** | Low | Low | Low | Low | Some Concern | Some Concern |
| **Zhang,2024** | Low | Low | Low | Low | Some Concern | Low |
|  |  |  |  |  |  |  |

| Supplement 10: PRISMA checklist | | | | | |
| --- | --- | --- | --- | --- | --- |
| **Section and Topic** | | **Item #** | | **Checklist item** | **Location where item is reported** |
| **TITLE** |  | |  |  |  |
| Title | | 1 | | Identify the report as a systematic review. | 1 |
| **ABSTRACT** | | | | |  |
| Abstract | | 2 | | See the PRISMA 2020 for Abstracts checklist. | 2 |
| **INTRODUCTION** | | | | |  |
| Rationale | | 3 | | Describe the rationale for the review in the context of existing knowledge. | 3 |
| Objectives | | 4 | | Provide an explicit statement of the objective(s) or question(s) the review addresses. | 4 |
| **METHODS** | | | | |  |
| Eligibility criteria | | 5 | | Specify the inclusion and exclusion criteria for the review and how studies were grouped for the syntheses. | 4 |
| Information sources | | 6 | | Specify all databases, registers, websites, organisations, reference lists and other sources searched or consulted to identify studies. Specify the date when each source was last searched or consulted. | 4 |
| Search strategy | | 7 | | Present the full search strategies for all databases, registers and websites, including any filters and limits used. | 5 |
| Selection process | | 8 | | Specify the methods used to decide whether a study met the inclusion criteria of the review, including how many reviewers screened each record and each report retrieved, whether they worked independently, and if applicable, details of automation tools used in the process. | 5 |
| Data collection process | | 9 | | Specify the methods used to collect data from reports, including how many reviewers collected data from each report, whether they worked independently, any processes for obtaining or confirming data from study investigators, and if applicable, details of automation tools used in the process. | 5 |
| Data items | | 10a | | List and define all outcomes for which data were sought. Specify whether all results that were compatible with each outcome domain in each study were sought (e.g. for all measures, time points, analyses), and if not, the methods used to decide which results to collect. | 5 |
|  |  | 10b | | List and define all other variables for which data were sought (e.g. participant and intervention characteristics, funding sources). Describe any assumptions made about any missing or unclear information. | 5 |
| Study risk of bias assessment | | 11 | | Specify the methods used to assess risk of bias in the included studies, including details of the tool(s) used, how many reviewers assessed each study and whether they worked independently, and if applicable, details of automation tools used in the process. | 5 |
| Effect measures | | 12 | | Specify for each outcome the effect measure(s) (e.g. risk ratio, mean difference) used in the synthesis or presentation of results. | 5 |
| Synthesis methods | | 13a | | Describe the processes used to decide which studies were eligible for each synthesis (e.g. tabulating the study intervention characteristics and comparing against the planned groups for each synthesis (item #5)). | 5 |
|  |  | 13b | | Describe any methods required to prepare the data for presentation or synthesis, such as handling of missing summary statistics, or data conversions. | 5 |
|  |  | 13c | | Describe any methods used to tabulate or visually display results of individual studies and syntheses. | 4 |
|  |  | 13d | | Describe any methods used to synthesize results and provide a rationale for the choice(s). If meta-analysis was performed, describe the model(s), method(s) to identify the presence and extent of statistical heterogeneity, and software package(s) used. | 4 |
|  |  | 13e | | Describe any methods used to explore possible causes of heterogeneity among study results (e.g. subgroup analysis, meta-regression). | 5,6 |
|  |  | 13f | | Describe any sensitivity analyses conducted to assess robustness of the synthesized results. | 5,6 |
| Reporting bias assessment | | 14 | | Describe any methods used to assess risk of bias due to missing results in a synthesis (arising from reporting biases). | 5,6 |
| Certainty assessment | | 15 | | Describe any methods used to assess certainty (or confidence) in the body of evidence for an outcome. | 6 |
| **RESULTS** | | | | |  |
| Study selection | | 16a | | Describe the results of the search and selection process, from the number of records identified in the search to the number of studies included in the review, ideally using a flow diagram. | 6 |
|  |  | 16b | | Cite studies that might appear to meet the inclusion criteria, but which were excluded, and explain why they were excluded. | 5,6 |
| Study characteristics | | 17 | | Cite each included study and present its characteristics. | 6 |
| Risk of bias in studies | | 18 | | Present assessments of risk of bias for each included study. | Supplement 9 |
| Results of individual studies | | 19 | | For all outcomes, present, for each study: (a) summary statistics for each group (where appropriate) and (b) an effect estimate and its precision (e.g. confidence/credible interval), ideally using structured tables or plots. | Supplement 2,3 |
| Results of syntheses | | 20a | | For each synthesis, briefly summarise the characteristics and risk of bias among contributing studies. | 6 |
|  |  | 20b | | Present results of all statistical syntheses conducted. If meta-analysis was done, present for each the summary estimate and its precision (e.g. confidence/credible interval) and measures of statistical heterogeneity. If comparing groups, describe the direction of the effect. | 6,7,8 |
|  |  | 20c | | Present results of all investigations of possible causes of heterogeneity among study results. | 6,7,8 |
|  |  | 20d | | Present results of all sensitivity analyses conducted to assess the robustness of the synthesized results. | 6,7,8 |
| Reporting biases | | 21 | | Present assessments of risk of bias due to missing results (arising from reporting biases) for each synthesis assessed. | Supplement 4 |
| Certainty of evidence | | 22 | | Present assessments of certainty (or confidence) in the body of evidence for each outcome assessed. | Supplement 5 |
| **DISCUSSION** | | | | |  |
| Discussion | | 23a | | Provide a general interpretation of the results in the context of other evidence. | 9,10,11 |
|  |  | 23b | | Discuss any limitations of the evidence included in the review. | 9,10,11 |
|  |  | 23c | | Discuss any limitations of the review processes used. | 9,10,11 |
|  |  | 23d | | Discuss implications of the results for practice, policy, and future research. | 9,10,11 |
| **OTHER INFORMATION** | | | | |  |
| Registration and protocol | | 24a | | Provide registration information for the review, including register name and registration number, or state that the review was not registered. | 3 |
|  |  | 24b | | Indicate where the review protocol can be accessed, or state that a protocol was not prepared. | 3 |
|  |  | 24c | | Describe and explain any amendments to information provided at registration or in the protocol. | 3 |
| Support | | 25 | | Describe sources of financial or non-financial support for the review, and the role of the funders or sponsors in the review. | 1,2 |
| Competing interests | | 26 | | Declare any competing interests of review authors. | 1 |
| Availability of data, code and other materials | | 27 | | Report which of the following are publicly available and where they can be found: template data collection forms; data extracted from included studies; data used for all analyses; analytic code; any other materials used in the review. |  |
